# Health related quality of life in adults after burn injuries: A systematic review

**DOI:** 10.1371/journal.pone.0197507

**Published:** 2018-05-24

**Authors:** Inge Spronk, Catherine Legemate, Irma Oen, Nancy van Loey, Suzanne Polinder, Margriet van Baar

**Affiliations:** 1 Association of Dutch Burn Centres, Maasstad Hospital, Rotterdam, the Netherlands; 2 Department of Public Health, Erasmus Medical Centre, Rotterdam, the Netherlands; 3 Department of Plastic, Reconstructive and Hand Surgery, Amsterdam Movement Sciences, VU University Medical Centre, Amsterdam, the Netherlands; 4 Burn Centre, Maasstad Hospital, Rotterdam, the Netherlands; 5 Association of Dutch Burn Centres, Red Cross Hospital, Beverwijk, the Netherlands; 6 Utrecht University, Department of Clinical Psychology, Utrecht, The Netherlands; National Natural Science Foundation of China, CHINA

## Abstract

**Objectives:**

Measurement of health-related quality of life (HRQL) is essential to qualify the subjective burden of burns in survivors. We performed a systematic review of HRQL studies in adult burn patients to evaluate study design, instruments used, methodological quality, and recovery patterns.

**Methods:**

A systematic review was performed. Relevant databases were searched from the earliest record until October 2016. Studies examining HRQL in adults after burn injuries were included. Risk of bias was scored using the Quality in Prognostic Studies tool.

**Results:**

Twenty different HRQL instruments were used among the 94 included studies. The Burn Specific Health Scale–Brief (BSHS-B) (46%), the Short Form–36 (SF-36) (42%) and the EuroQol questionnaire (EQ-5D) (9%) were most often applied. Most domains, both mentally and physically orientated, were affected shortly after burns but improved over time. The lowest scores were reported for the domains ‘work’ and ‘heat sensitivity’ (BSHS-B), ‘bodily pain’, ‘physical role limitations’ (SF-36), and ‘pain/discomfort’ (EQ-5D) in the short-term and for ‘work’ and ‘heat sensitivity’, ‘emotional functioning’ (SF-36), ‘physical functioning’ and ‘pain/discomfort’ in the long-term. Risk of bias was generally low in outcome measurement and high in study attrition.

**Conclusion:**

Consensus on preferred validated methodologies of HRQL measurement in burn patients would facilitate comparability across studies, resulting in improved insights in recovery patterns and better estimates of HRQL after burns. We recommend to develop a guideline on the measurement of HRQL in burns. Five domains representing a variety of topics had low scores in the long-term and require special attention in the aftermath of burns.

## Introduction

Surviving a severe burn injury is considered a traumatic experience. Due to substantial improvements in burn treatment, an increasing number of patients survive burns [1, 2]. This increases the importance of documenting outcomes of burns on both the short- and long-term as a significant number of patients face physical and/or psychological consequences, such as post-traumatic stress symptoms, depression, and limited physical functioning [3–5]. Moreover, disabilities and disfigurement are frequently accompanied with burn injury.

Health related quality of life (HRQL) is an outcome measure that reflects a patient’s perception of his or her health condition on physical, psychological and social wellbeing after an injury or disease [6]. In general, HRQL is assessed by questionnaires filled in by patients. HRQL instruments are either generic (i.e. applicable to any illness) or disease-specific. Generic instruments facilitate comparison between different diseases, whereas burn-specific instruments take the specific effects of burns into account [7]. HRQL measurement is increasingly used in both clinical practice and burn research to qualify the impact of burns [3, 8]. It may help to tailor aftercare to the patient’s need.

Although, some earlier reviews on the HRQL of burn patients have been performed, there is no recent systematic review on this topic. Yoder et al. conducted a systematic review on the evolution of one burn-specific HRQL instrument; the burn specific health scale (BSHS) [9]. Outcomes were, however, not reported. Stavrou et al. only provided a narrative overview of the domains that could be impaired after burns [10, 11].

In conclusion, there is a need for a systematic review to identify which HRQL instruments are used in burns and to examine recovery patterns after burns. Therefore, the aims of this review are 1) to identify which generic and burn specific instruments are used for the measurement of HRQL after burn injuries in adults and 2) to examine recovery patterns of HRQL after burns.

## Methods

The present review was conducted and reported in line with the Preferred Reporting Items for Systematic Reviews and Meta-analyses (PRISMA) Statement [12]. The protocol for this systematic review was registered on PROSPERO (ID = CRD42016048065) and is available online (http://www.crd.york.ac.uk/PROSPERO/display_record.asp?ID=CRD42016048065).

### Search strategy and eligibility criteria

A systematic search using terms covering HRQL and burns (S1 File) was conducted from the earliest record until October 2016. The search strategy was developed in collaboration with a librarian with extensive experience in systematic reviews. The databases included Embase, Medline, CINAHL, Cochrane, Web of Science and Google scholar. Original research studies conducted in adult burn patients, written in English and published in a peer-reviewed journal were included. Studies were required to have a generic or disease-specific HRQL as outcome measure and burn patients had to be treated at a health care facility. This includes patients that required inpatient hospitalisation, but also patients treated at an emergency department as well as in outpatient care. Studies that included data on other patient groups, in addition to burn patients, and that not present HRQL outcomes for burn patients separately were excluded.

### Selection of studies and data extraction

After removal of duplicates, articles were excluded on the basis of title by one reviewer (IS). Two reviewers (IS and CL) independently evaluated a random sample of ten percent of the abstracts. As there was no disagreement between the reviewers, the remaining abstracts were appraised by one reviewer (IS). In case of any doubt, a title or abstract was screened by a second reviewer. Screening of full texts and extraction of data was done independently by two researchers (IS and CL). The titles, abstracts or full texts were evaluated using the inclusion criteria described above. Extracted information included study characteristics, patient characteristics, details on the instruments used to assess HRQL and HRQL outcomes at each assessment point. Disagreements around article inclusion or extraction of data were resolved by discussion with a third researcher (MvB).

### Risk of bias assessment

The risk of bias of all eligible studies was assessed using four of the six domains of the Quality in Prognostic Studies (QUIPS) risk of bias tool [13]. We included the domains: study participation, study attrition, outcome measurement and statistical analysis and presentation. Two domains ‘prognostic factor measurement’ and ‘study confounding’ were not included as these domains are specific for prognostic studies and thus fell outside the scope of the review. The domains were rated as ‘low’ bias (all items ‘low risk’), ‘moderate’ bias (max. 50% items with high or unknown risk of bias) or ‘high’ risk of bias (>50% items high of unknown risk of bias).

First, two researchers (IS and CL) were trained to use the QUIPS and independently assessed the risk of bias of eighteen eligible studies (19%)). Discrepancies were discussed with a third researcher (MvB). Then, the researchers independently assessed a random sample of 25 of the remaining articles (33%). There was only a slight disagreement (7%) and therefore the remaining studies were appraised by one researcher (IS). In case of any doubt, a study was appraised by a second reviewer.

### Data analysis

In case of multiple studies using an identical dataset, the study that included the most assessment points, the most patients or the most HRQL domains was chosen. If no decision could be made, the most recent publication was selected.

If scores were only presented in figures, authors were asked to provide the scores. If authors did not respond, the scores were read from the graph and were rounded to the nearest 0.5 points. If domain scores were only presented as norm scores, authors were asked to provide the non-normalized domain scores. If no scores were received, the outcomes were not included in the recovery pattern analyses. Outcomes of studies were only included when the study population included at least 10 patients.

## Results

### Identification and selection of studies

**Fig 1:**
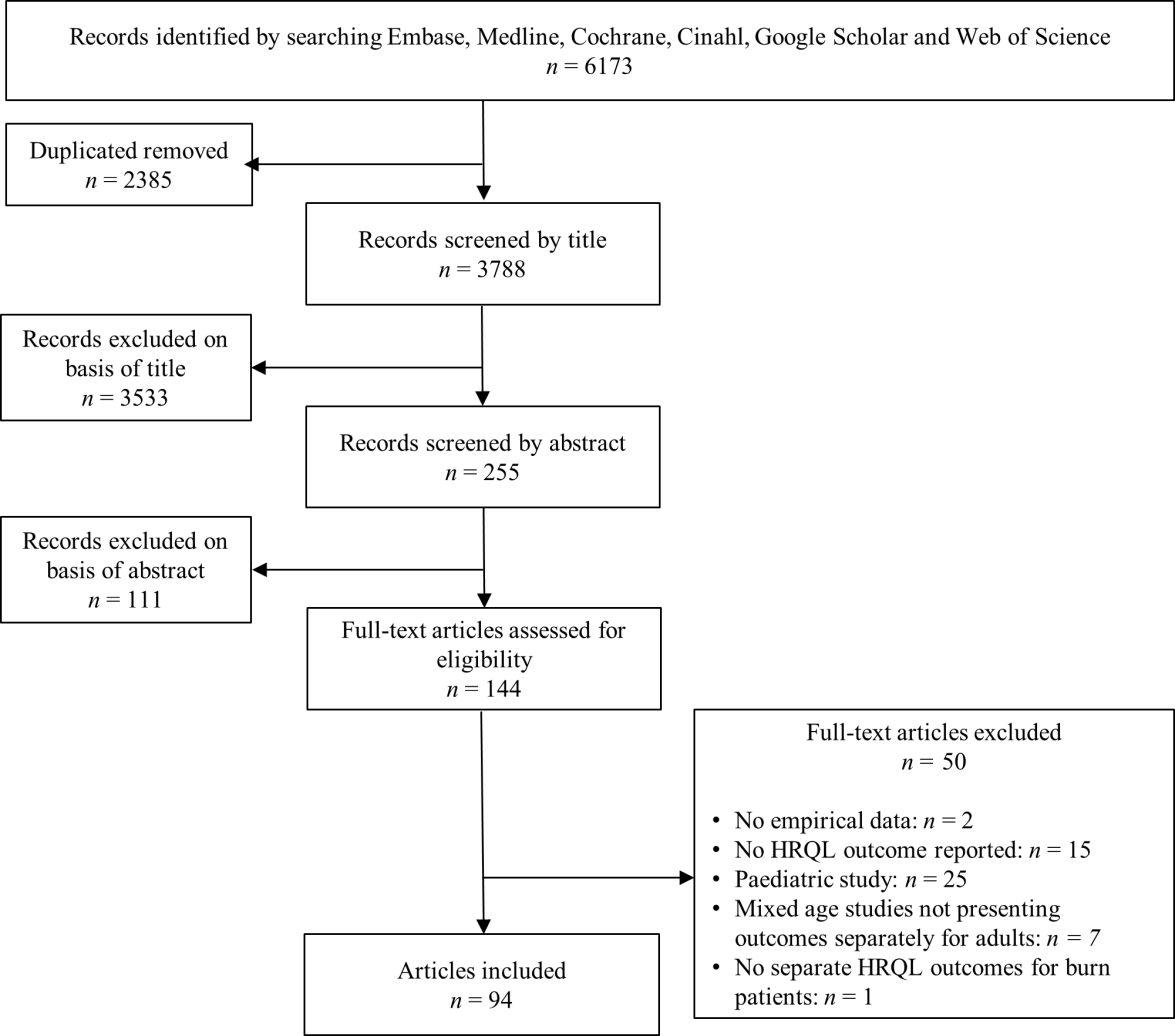
Flowchart outlining selection of studies.

The search resulted in 3,788 unique articles. Screening of titles resulted in 255 potentially relevant articles. Of these, 111 were excluded on the basis of abstract and 144 were retrieved for full-text review (Fig 1). Fifty-one of these articles did not meet all inclusion criteria, resulting in the inclusion of 94 articles (S1 Table).

### Study characteristics

Most studies were conducted in Europe (n = 37), the USA (n = 19) and Australia (n = 14). More than half (n = 54) of the studies were published after 2010 and most had a cross-sectional design (n = 57) (Table 1). Sample sizes of the studies varied between 9 [14] and 1,587 [15] burn patients, with most studies having a sample size below 200 patients (86%). In most studies (n = 83) more males than females were included, although not all studies provided details on the sex distribution (n = 6) [15–20]. The mean %TBSA burned ranged from 3.5% [21] to 83.5% [22]. Eight studies did not report the %TBSA burned of the included patients. Mean LOS was between 10 and 30 days in most studies. In total, 35 studies failed to report the mean length of stay.

**Table 1:** Study characteristics of 94 studies measuring HRQL in adult burn patients.

Study characteristics		Studies (n)	References
**Study type**			
	Case-control	3	[14, 21, 23]
	Cohort	32	[15, 20, 24–52]
	Cross-sectional	56	[10, 16–19, 22, 53–101]
	Trial	3	[102–104]
**Patient sample size**			
	0–20	7	[14, 22, 40, 48, 58, 81, 87]
	>20–50	19	[16–18, 21, 25, 33, 34, 51, 54, 55, 59, 60, 66, 71, 72, 83, 84, 96, 99]
	>50–100	30	[19, 28, 30, 31, 36–39, 44, 46, 50, 52, 53, 56, 57, 62, 64, 74, 77–80, 85, 88, 90, 92, 97, 104, 105]
	>100–200	25	[24, 29, 35, 42, 43, 47, 49, 61, 63, 65, 68–70, 75, 76, 82, 89, 91, 93–95, 100, 102, 103, 106]
	>200–500	9	[26, 41, 45, 67, 73, 86, 98, 101, 107]
	>500	3	[15, 27, 32]
	NA	1	[20]
**Mean %TBSA burned**			
	0–10%	9	[21, 26, 27, 30, 39, 51, 66, 71, 86]
	>10–20%	32	[28, 29, 31, 40–48, 53, 60, 63, 70, 76–80, 82, 84, 88, 90, 94, 95, 97, 101, 102, 105, 106]
	>20–30%	26	[17–19, 24, 25, 32, 35–38, 54, 58, 61, 64, 65, 67–69, 72, 74, 85, 91, 96, 99, 103] [52]
	>30–40%	6	[16, 23, 34, 73, 81, 89]
	>40–50%	5	[14, 83, 93, 98, 107]
	>50–60%	4	[56, 57, 87, 92]
	>60–70%	2	[55, 59]
	>70–80%	0	-
	>80–90%	2	[22, 100]
	NA	8	[15, 20, 33, 49, 50, 62, 75, 104]
**Mean length of stay (days)**			
	0–10	2	[50, 71]
	>10–20	15	[21, 26, 31, 40, 47, 51, 53, 54, 60, 70, 86, 98, 103, 106] [46]
	>20–30	29	[19, 25, 29, 32, 34–38, 45, 49, 52, 61, 69, 74, 76–80, 84, 90, 94–97, 101, 102, 105]
	>30–40	8	[18, 44, 65, 67, 68, 72, 87, 99]
	>40–50	1	[58]
	>50–60	0	-
	>60–70	1	[104]
	>70–80	1	[55]
	>80	2	[22, 73]
	NA	35	[14–17, 20, 23, 24, 27, 28, 33, 39, 41–43, 48, 56, 57, 59, 62–64, 66, 75, 81, 83, 85, 88, 89, 92, 93, 100, 107]
**Number of HRQL instruments**			
	1 instrument	63	[16–21, 23–25, 28–35, 37, 38, 42–45, 50, 51, 53, 54, 58, 59, 62, 63, 66–72, 74, 75, 79, 81, 84–87, 89–95, 97–104, 106, 107]
	2 instruments	24	[14, 22, 26, 27, 40, 41, 46–49, 52, 55–57, 60, 64, 65, 78, 80, 82, 83, 88, 96, 105]
	3 instruments	7	[15, 36, 39, 61, 73, 76, 77]
**Number of assessment time points**			
	1 time point	61	[14, 16–19, 21–23, 39, 53–101, 104, 105, 107]
	2 time points	5	[28, 34, 35, 46, 102]
	3 time points	4	[24, 44, 52, 103]
	4 time points	12	[29, 30, 33, 36, 38, 42, 43, 45, 47–49, 51]
	5 time points	10	[15, 25–27, 32, 37, 40, 41, 50, 106]
	>5 time points	1	[31]
	NA	1	[20]

*Note*. NA = not applicable, TBSA = total body surface area

### Measurement of HRQL

**Fig 2:**
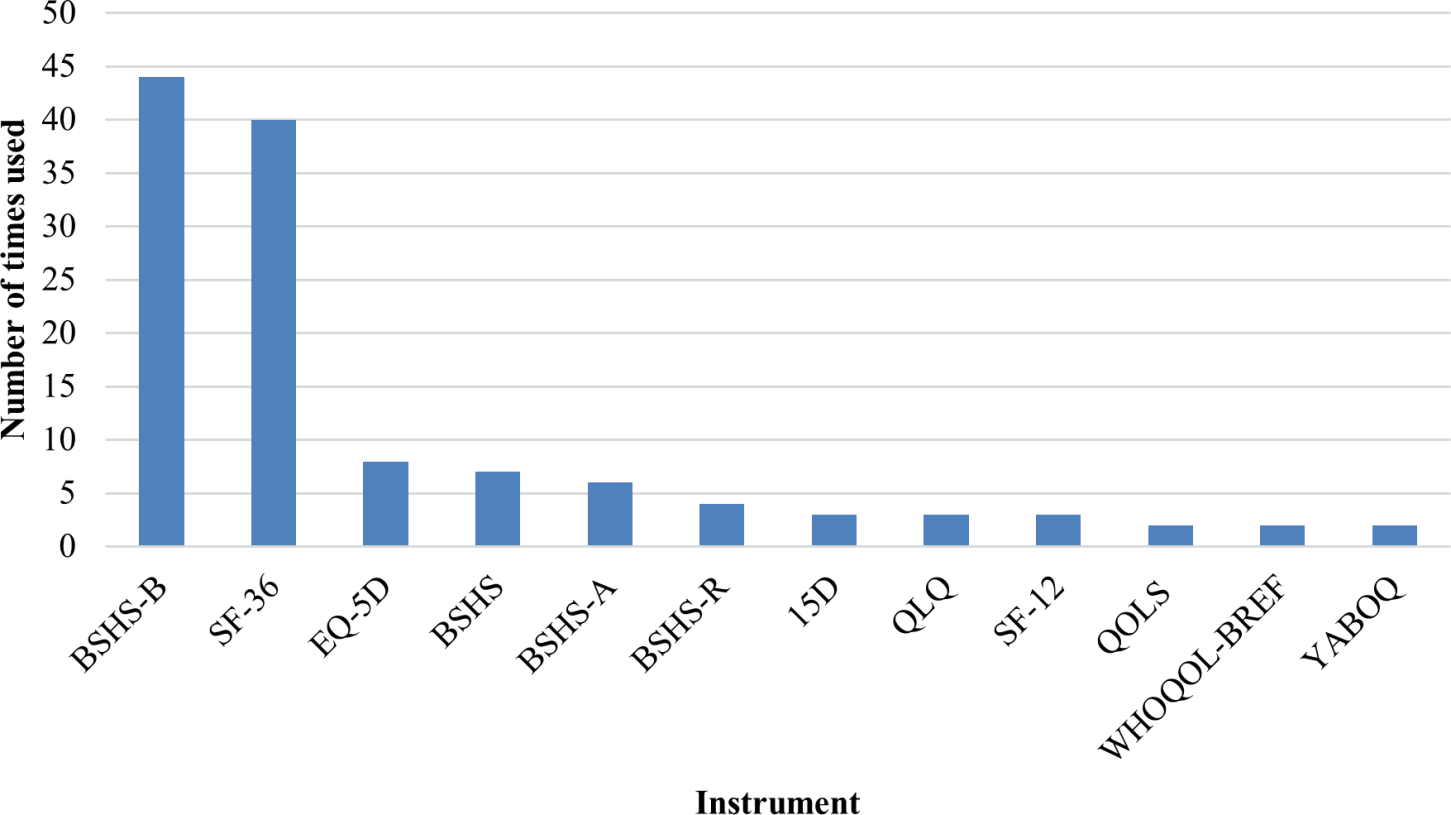
Instruments used to measure health-related quality of life in >1 study. BSHS-B = Burn Specific Health Scale—Brief, SF-36 = Medical Outcome Study Short Form—36 items, EQ-5D = EuroQol five dimensions questionnaire, BSHS = Burn Specific Health Scale, BSHS-A = Burn Specific Health Scale—Abbreviated, BSHS-R = Burn Specific Health Scale Revised, 15D = 15-dimensional health-related quality of life instrument, QLQ = Quality of Life Questionnaire, SF-12 = Medical Outcome Study Short Form—12 items, QOLS = Quality of Life Scale, WHOQOL-BREF = World Health Organization Quality of Life—BREF, YABOQ = Young Adult Burn Outcome Questionnaire.

**Fig 3:**
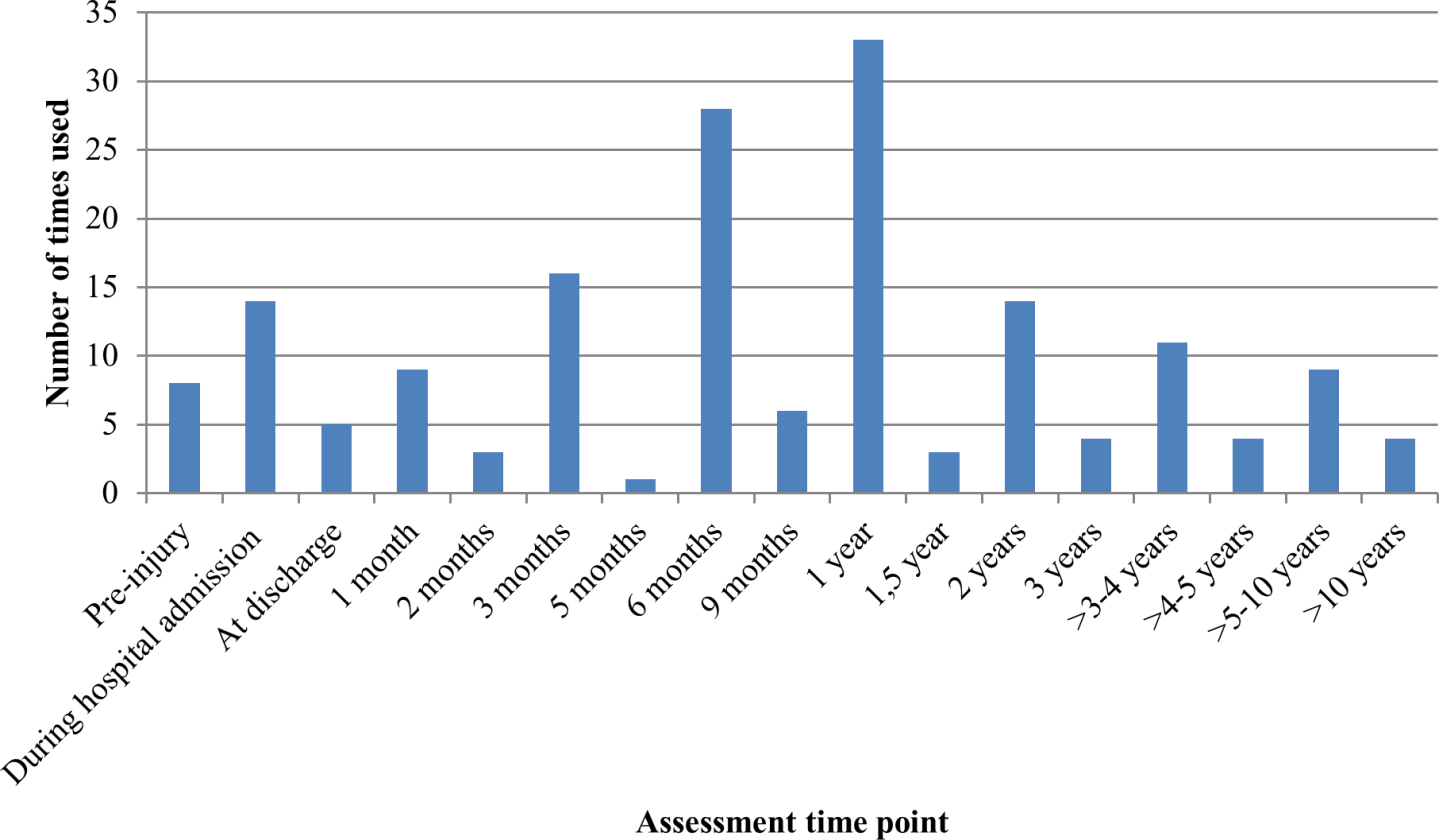
Time points at which health-related quality of life in burn patients was assessed. *Note*. Data on pre-burn HRQL is collected retrospectively.

Twenty different instruments, of which eight are validated in the burn population, were used to assess HRQL. The three most often applied instruments were the Burn Specific Health Scale—Brief (BSHS-B) (n = 44), the Medical Outcome Study Short Form—36 items (SF-36) (n = 40), and the EuroQol five dimensions questionnaire (EQ-5D) (n = 8) (Fig 2). Eight instruments were only used in one single study. Thirty-one studies used more than one instrument to assess the HRQL (Table 1). Twenty-four of these used a burn-specific and a generic HRQL instrument. Most used both the SF-36 and the BSHS-B (n = 18). Thirty-two studies (34%) used a longitudinal design with multiple HRQL assessments over time; twenty-three studies used at least four assessment points. The most frequently used assessment time points were during hospital admission, and at 3 months, 6 months, 12 months and at 24 months after injury (Fig 3).

### Quality assessment

**Fig 4:**
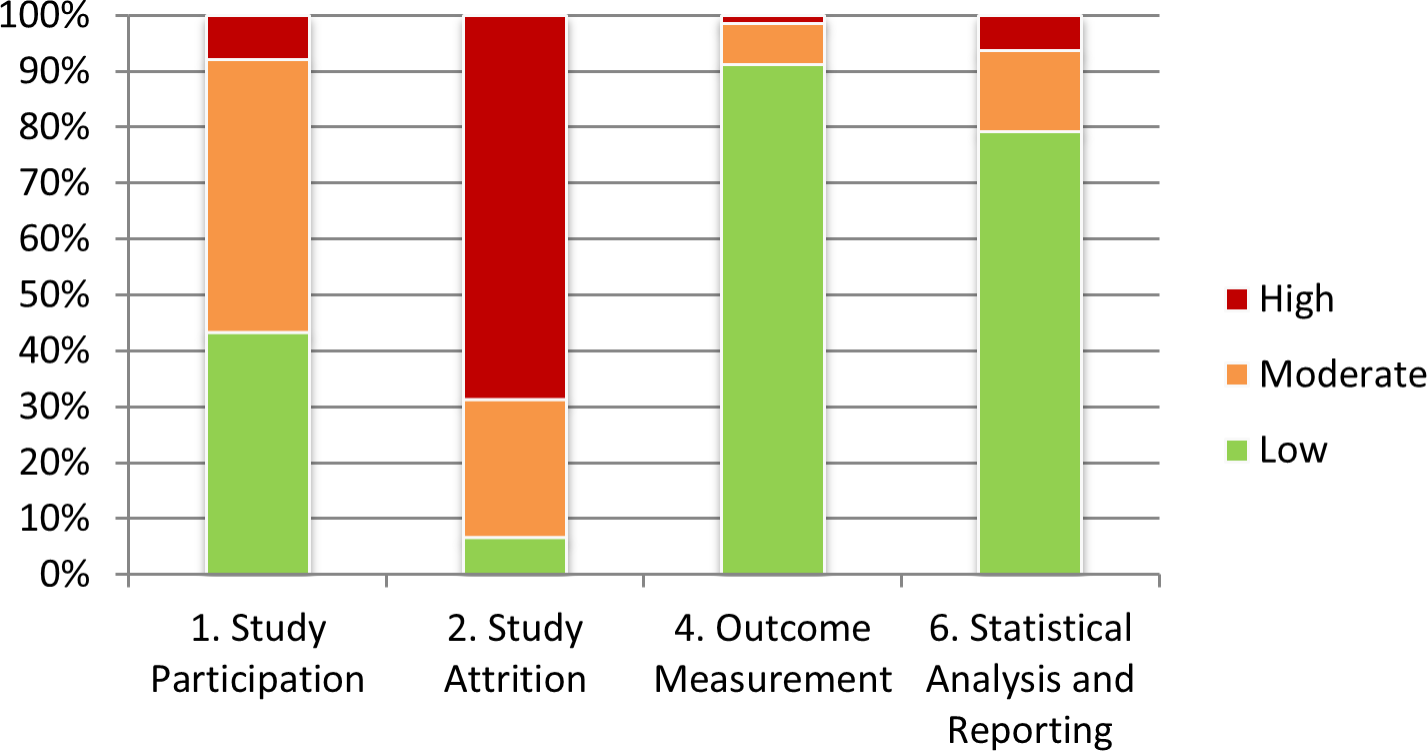
Risk of bias assessed with four domains of the Quality in Prognostic Studies (QUIPS) risk of bias tool.

The risk of bias was evaluated using the QUIPS tool. Whilst most studies had low risks of bias on ‘outcome measurement’ (n = 87) and ‘statistical analysis and reporting’ (n = 75), a moderate or high risk was evident in many studies for ‘study attrition’ (n = 88) (see Fig 4; S2 Table). This was in particular caused by a lack of reporting of attempts to collect information on drop-outs and of key characteristics of non-responders. Four studies [27, 32, 33, 54] scored a low risk of bias on all four evaluated items of the QUIPS.

### Recovery patterns of HRQL after burns in adults

Recovery patterns of the most applied instruments, the BSHS-B, the SF-36 and the EQ-5D, which are all validated within the burn population, were analysed. All studies that reported a BSHS-B or BSHS-R outcome, a SF-36 outcome or an EQ-5D outcome on at least one time point were included.

#### BSHS-B recovery patterns

The BSHS-B includes 40 items comprising nine HRQL domains: simple abilities, heat sensitivity, hand function, treatment of regimens, work, body image, affect, interpersonal relationships and sexuality [108]. Responses on individual items are scored on a five-point scale ranging from 0 (extremely) to 4 (not at all). Mean scores per domain were assessed and high scores refer to a good perceived health status. Of the 47 studies that used the BSHS-B or BSHS-R, 17 could be used to analyze HRQL recovery patterns [19, 26, 38, 40, 49, 60, 64, 67, 76, 81–83, 88, 90, 91, 102, 105] (S1 Fig).

**Fig 5:**
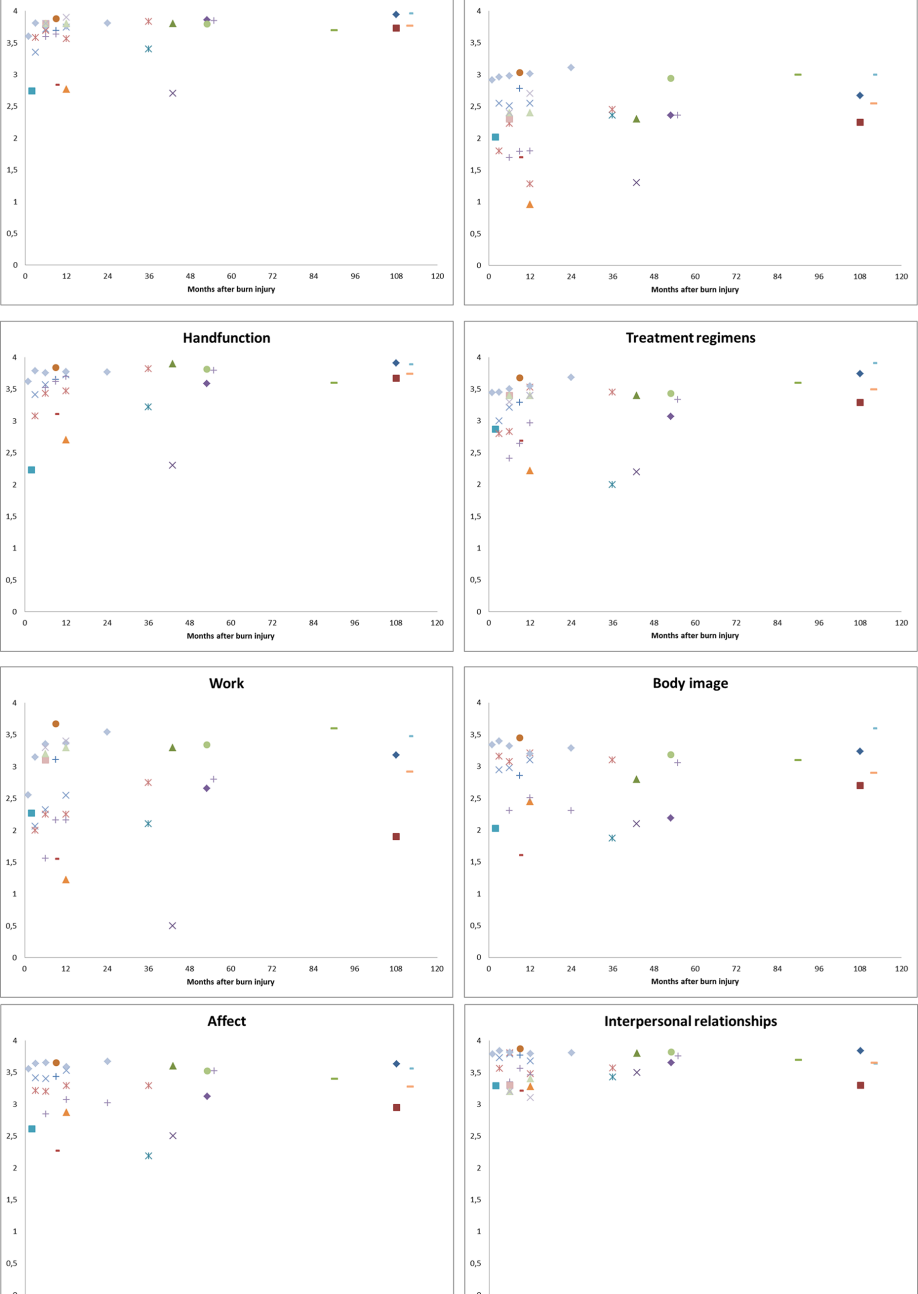
a. BSHS-B domain scores for six domains for seventeen studies. b. BSHS-B domain scores for three domains for seventeen studies.

Overall, shortly after burns, scores on the different domains were low and most increased with time (Fig 5A and 5B). In the short-term, most problems were reported for the domains ‘work’ and ‘heat sensitivity’. The self-reported outcomes of the domains ‘simple abilities’, ‘hand function’, ‘affect’, ‘heat sensitivity’, ‘body image’ and ‘treatment regimens’ showed improvement over time. Low scores were especially seen in the first 12 months after burns and improved afterwards. On average, outcomes of the domains ‘simple abilities’ and ‘hand function’, improved towards the maximum score, whereas the domains ‘affect’ and ‘treatment regimens’ improved to 3.5 out of 4, e domain ‘body image’ improved towards 3 out of 4 and the domain ‘heat sensitivity’ towards 2.5 out of 4. The domain ‘sexuality’ remained relatively stable, only few studies reported somewhat lower scores in the short-term. The outcomes of the ‘interpersonal relationships’ domain were relatively high during the entire follow-up. The self-reported outcomes of the last domain, the domain ‘work’, varied widely among studies. In general, subgroups with less severe problems (i.e. no surgery, no full thickness burn) had higher scores on all domains.

#### SF-36 recovery patterns

The SF-36 consists of 36 items comprising eight domains: physical functioning, role limitations-physical, bodily pain, general health, vitality, social functioning, and role limitations emotional, and mental health. Mean domain scores that were transformed to a 0 (the worst) to 100 (the best) scale were used. Higher scores indicate a greater perceived health. The SF-36 domains can be summarized into the physical component summary (PCS) and the mental component summary (MCS) [109]. These measures are transformed to norm-based scores with a mean of 50 and a standard deviation (SD) of 10. Scores lower than 50 indicate scores below the average. Analyses of recovery patterns of the SF-36 outcome data were based on 17 studies of the 40 studies that assessed HRQL with the SF-36 [23, 26, 28, 29, 32, 35, 40, 44, 49, 60, 64, 66, 72, 73, 76, 80, 99] (S2 Fig).

**Fig 6:**
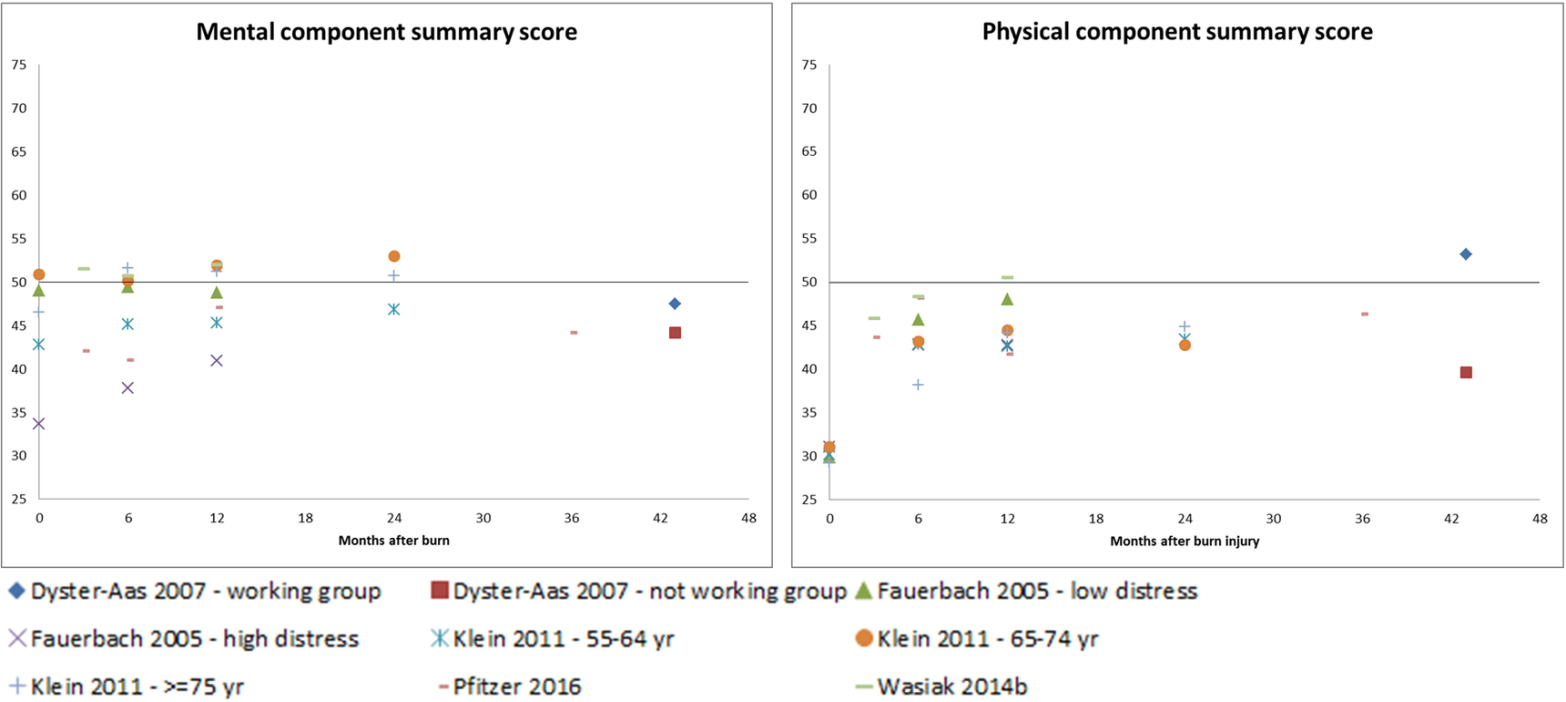
SF-36 physical component summary scores and mental component summary scores for five studies. The black line in the figures represents the US-norm score.

Four out of the 17 studies described all eight domains of the SF-36 as well as the PCS and MCS. Ten studies included the eight domains, one study included seven domains [76], and one study described both summary scores [32]. The MCS scores showed variation in the short-term, with studies reporting scores just above and below the norm score (Fig 6). In the longer-term, scores moved towards the norm score. PCS scores were almost all below the norm score and an improvement towards the norm was seen in the longer-term.

**Fig 7:**
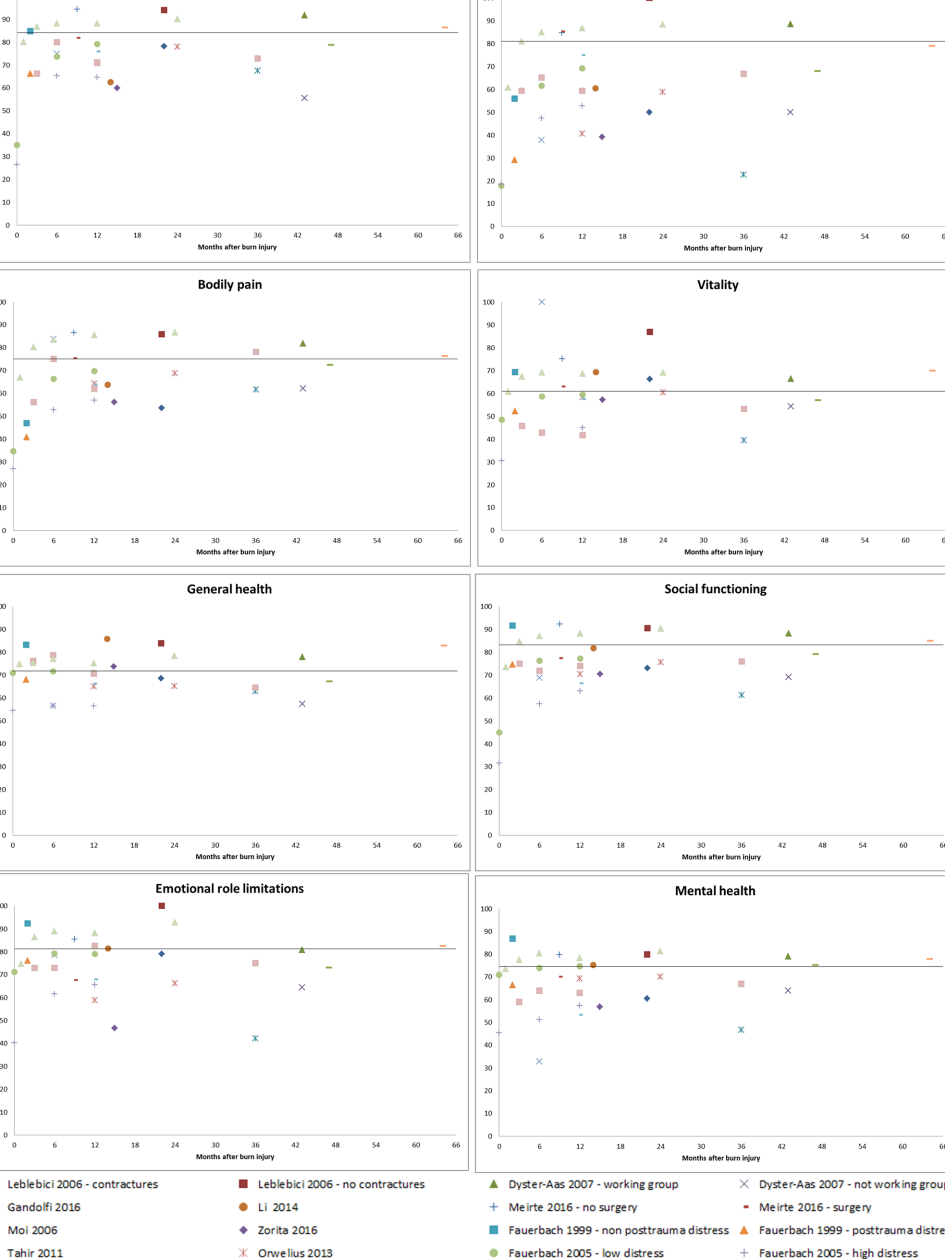
a. SF-36 domain scores for six dimensions for fourteen studies. The line in the figures represent the US-norm score. b. SF-36 domain scores for two dimensions for fourteen studies. The line in the figures represent the US-norm score.

The lowest scores were reported for the domains ‘bodily pain’ and ‘physical role limitations’ in the short-term and for the domains ‘physical role limitations’ and ‘emotional role limitations’ in the longer-term (Fig 7A and 7B). Four domains, including ‘physical functioning’, ‘bodily pain’, ‘social functioning’ and ‘mental health’, showed a similar pattern with lower scores shortly after burns and these improved towards the norm afterwards. The other four domains showed different patterns. The domain ‘vitality’ showed a large variety in obtained scores in the short-term, both below and above the US-norm score. However, afterwards, scores were closer to the norm score. The self-reported outcomes of the ‘general health’ domain remained constant during the whole follow-up time. Scores of the domain ‘emotional role limitations’ were relatively high shortly after burns, but lower scores were reported in the longer-term. The outcomes of the remaining domain, ‘physical role limitations’, varied widely among studies during the entire follow-up period. Overall, subgroups with less severe injury (i.e. no surgery, no contractures) had higher scores on all domains.

#### EQ-5D recovery patterns

The EQ-5D consists of five dimensions: mobility, self-care, usual activities, pain/discomfort and anxiety/depression and a visual analogue scale (VAS) for general health. Each dimension has three levels of severity: no problems, moderate problems or severe problems [110]. Based on the answers of the five dimensions, a single index value can be derived ranging from 0 (death) to 1 (full health). Eight studies used the EQ-5D; data of 5 studies could be used to examine the recovery patterns based on the EQ-5D. Three studies were based on the same data source as studies already included in the analyses and were therefore not used [36, 61, 76]. As only two studies included a time point after 12 months (resp. 18 months [45] and on average 55 months [37]), no firm conclusions can be drawn on longer-term recovery.

**Fig 8:**
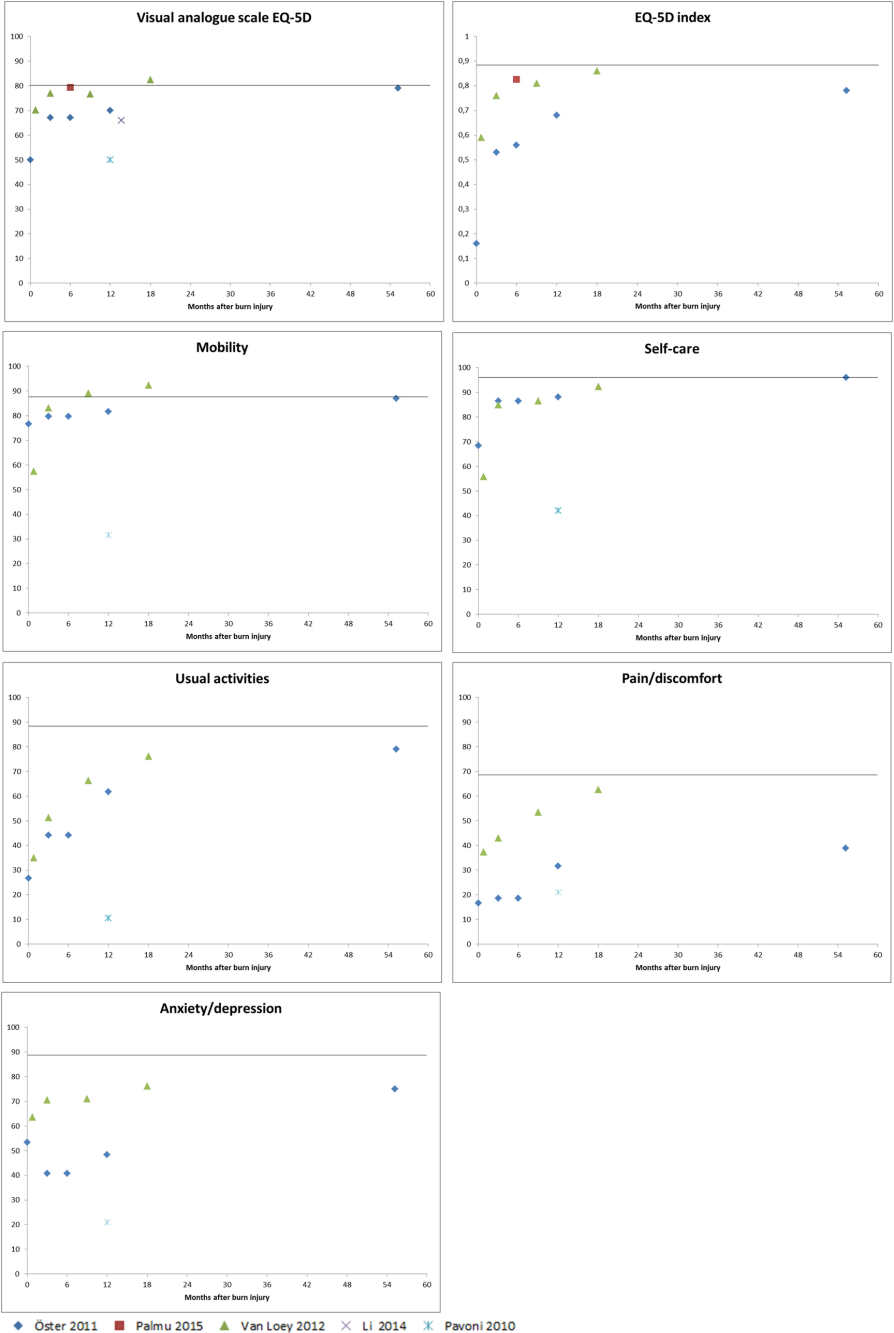
EQ-5D scores the visual analogue scale, the EQ-5D index and five dimensions for three to five studies. The line in the figures represent the composed norm score based on norm scores of the countries where the studies were conducted [111]. The y-axis represents 0–100% patients with no problems on a specific domain.

All studies reported the EQ-5D VAS score for general health. Reported scores were lower shortly after burns and increased with time towards the norm score (Fig 8). The study reporting lower scores at 12 months was the only study in more severe burn patients [87]. Lowest scores shortly after burns were seen for the EQ-5D index and the ‘pain/discomfort’ domain. The EQ-5D VAS score improved towards the norm score in the longer-term, just as the ‘mobility’ and ‘self-care’ domain. The self-reported outcomes of two other domains, 'usual activities', and 'anxiety/depression' and the EQ-5D index showed some improvement over time, but did not reach the level of the norm scores. The outcomes of the last domain 'pain/discomfort' did not show much improvement over time.

## Discussion

This review provides a comprehensive overview of generic and burn specific instruments used to measure HRQL in adult burn patients and examined recovery patterns of HRQL in burns. Twenty HRQL instruments were used among the 94 studies. The BSHS-B and the SF-36 were most widely applied followed by the EQ-5D. It was seen that scores on most domains, both mental and physically orientated, were lower shortly after burns and improved over time. However, the BSHS-B domains 'work' and ‘heat sensitivity’, the SF-36 domains 'emotional role limitations' and 'physical role limitations', and the EQ-5D domain ‘pain/discomfort’ showed considerable variation across studies and low scores were also reported in the longer-term. The methodological quality of the included papers was in general moderate.

This review showed that there is some agreement on instruments used for the measurement of HRQL in adults after burns. Both instruments that are validated and that are not validated in the burn population are used. The majority of studies (70%) used the BSHS-B, the SF-36, or a combination of both instruments and eight studies (9%) used the EQ-5D, which are all validated in the burn population. It is recommended to use both a validated generic and burn specific instrument to assess the HRQL to capture the full impact of a health condition [112]. However, only 24 (26%) of the included studies used a combination of instruments. The (additional) use of a generic instrument, the SF-36 or the EQ-5D has the advantage that norm scores are available. The use of norm scores facilitates the comparison with other populations and interpretation of the outcomes. For the BSHS-B, partial population norm scores are available, including 30 of 40 items of the BSHS-B; the remaining ten items were considered too specific for burns [113]. Unfortunately, the results are not summarized on domain level. This would have provided norm scores for six of the BSHS-B domains. In the absence of population norm scores, domain scores reported by burns survivors in the long-term can be used as norm values.

Despite the widespread use of the BSHS-B, there is discussion about this instrument. A study comparing the SF-36 with the BSHS-B found that the SF-36 domains are more sensitive than the BSHS-B domains from 1 month post burn [26]. Besides, there is no evidence on test-retest ability, validity of hypothesis testing and item-total correlations of the BSHS-B [114]. Currently several new instruments are being developed by different research groups [114–117], resulting in different instruments which may hamper the comparison of outcomes in the future. There is a need to achieve consensus on which HRQL instruments are best to use in burn populations and at which time points. The studies with a longitudinal design (n = 32) showed overlap in their assessment points. Most studies assessed HRQL at baseline, 3 months, 6 months, 12 months and 24 months post burn. Given the high attrition rates in burn studies, it may be difficult to obtain longer follow-up. However, a further improvement of HRQL beyond this period may be expected as it is known that HRQL further improves after 24 months [37, 38].

The three HRQL questionnaires have overlapping domains [118]. For example, the domains ‘simple abilities’ (BSHS-B), ‘physical functioning’ (SF-36), ‘mobility’ (EQ-5D) and ‘self-care’ (EQ-5D) all measure activity limitations. Results on the different questionnaires show congruent results; activity is limited shortly after burns and improves with time. This is in line with the course of the recovery of burns as shortly after burns wounds are healing and physical capability is impaired. When wounds are healed activity improves. However, participation restrictions due to physical functioning are seen in both the short- and longer-term. The three domains covering this (‘work’ (BSHS-B), ‘physical role limitations’ (SF-36), and ‘usual activities’ (EQ-5D)) show mixed results, with also reduced scores in the longer-term. Simple activities like walking and dressing improve towards the level of the average population, however, more advanced functioning like working is more affected by burns and varies among the population, which might be explained by the heterogeneous nature of the burn population in combination with reported substantial effects on work situation, also in burns of limited severity [119].

Participation restrictions due to emotional and mental well-being (‘interpersonal relationships’ (BSHS-B), ‘social functioning’ (SF-36) and ‘emotional role limitations’ (SF-36)) are less prevalent after burns. In the short term there are some limitations with social activities, but this improves over time. In the longer-term, limitations of regular daily activities, including work, because of emotional problems seem to develop. Patients accomplish less than they would like and work not as carefully as usual.

Mental function improved over time. This was consistent across the questionnaires (‘affect’ (BSHS-B), ‘mental health’ (SF-36) and ‘anxiety/depression’ (EQ-5D)). However, the scores for anxiety and depression did not reached the level of the general population, indicating that burn patients are on average more anxious or depressed.

Results on pain varied between the domains measuring this construct. According to the ‘bodily pain’ domain of the SF-36, the level of pain decreases with time and is comparable to the level of the general population in the longer-term, whereas the domain ‘pain/discomfort’ from the EQ-5D shows that the majority of patients experience pain or other discomfort in the longer-term. This is a much higher percentage than the proportion of the general population experiencing pain. Pain might thus be an issue in some patients in the longer-term, but does not seem to interfere with daily activities.

This review has a number of strengths and limitations. Strengths include the comprehensive overview of HRQL instruments used in burn populations, based on six databases, and the identification of HRQL domains that need more attention in the aftermath of burn injuries. However, some limitations also merit note. The scope of the review was limited to English-language studies, which might have resulted in missed studies that were published in foreign language journals. Another limitation is the wide variation in both study designs and instruments used, impeding a meta-analysis. Besides, due to the low number of longitudinal studies, we had to use cross-sectional studies to examine recovery patterns. Also the review was hampered by different ways of reporting the results, including mean or median scores, domain scores versus total scores and 0–100 scores or standardized norm scores, which makes it hard to compare results. Besides, the methodological quality of included studies varied widely. The most alarming was the general high risk of bias on study attrition. Only few studies adequately reported attempts to collect information on participants who dropped out and key characteristics on those lost to follow-up. In future articles it is important to include description of these factors in order to reach a low risk of bias on study attrition and improve the overall study quality.

## Conclusion

This review demonstrates that most domains of HRQL, frequently measured using the BSHS, SF-36 or EQ-5D, are affected shortly after the burn event. Most domains will recover over time excluding physical and emotional role participation, anxiety, depression and pain. This reflects the need for both mental and physical support in the aftermath of burns. To further facilitate the comparability of burn-related HRQL outcomes across the world, use of uniform validated instruments, time points and data presentation is needed. It is therefore recommended to develop a guideline on the measurement of HRQL in burn patients.

## Supporting information

S1 File Search strategy. (DOCX)

S2 File Extraction sheet HRQL review. (XLSX)

S1 Table Characteristics of studies. ^1^Study population: n = sample size; M = males; NA = not applicable. ^2^15D = 15-dimensional health-related quality of life instrument, ALLTAGSLEBEN = multidimensional German questionnaire "every-day-life", BSHS = Burn-specific Health Scale, BSHS-A = Burn-specific Health Scale—Abbreviated, BSHS-B = Burn-specific Health Scale—Brief, BSHS-R = Burn-specific Health Scale—Revised, BSHS-RBA = Burn-specific Health Scale Revised, Brief and Adapted, DLQI = Dermatology Life Quality Index, EQ-5D = EuroQol five dimensions questionnaire, RAND-36 = RAND 36-item health survey, SIP = Sickness Impact Profile, SF-8 = Medical Outcome Study Short Form—8 items, SF-10 = Medical Outcome Study Short Form—10 items, SF-12 = Medical Outcome Study Short Form—12 items, SF-36 = Medical Outcome Study Short Form—36 items, QLQ = Quality of Life Questionnaire, QOLS = quality of life scale, WHODAS = World Health Organization Disability Assessment Schedule, WHOQOL-BREF = World Health Organization Quality of Life—BREF, YABOQ = Young Adult Burn Outcome Questionnaire.
(DOCX)

S2 Table Risk of bias assessed with four domains of the Quality in Prognostic Studies (QUIPS) risk of bias tool. (DOCX)

S1 Fig Flowchart BSHS-B studies. (TIF)

S2 Fig Flowchart SF-36 studies. (TIF)
