# Supplementary material for: Health related quality of life in adults after burn injuries: A systematic review
Source: PLoS One. 2018 May 24;13(5):e0197507. doi: 10.1371/journal.pone.0197507 (PMC5967732; doi:10.1371/journal.pone.0197507)
Supplement: S1 File — (DOCX) [file pone.0197507.s001.docx]

**S1 File . Search strategy**

Embase.com

('thermal injury'/de OR 'heat injury'/de OR 'burn'/exp OR 'burn patient'/de OR 'burn scar'/de OR 'burn nursing'/de OR 'burn unit'/de OR (((burn OR burned ) NOT burn*-out*) OR burns OR ((thermal OR heat) NEAR/3 injur*)):ab,ti) AND ('quality of life'/exp OR 'health status'/exp OR 'general health status assessment'/exp OR 'health status indicator'/de OR 'disability'/exp OR 'work resumption'/de OR 'return to work'/de OR 'sexual function'/de OR 'daily life activity'/exp OR 'chronic pain'/exp OR 'functional disease'/de OR 'functional assessment'/de OR 'disabled person'/de OR 'physical activity'/exp OR 'physical performance'/exp OR 'independent living'/exp OR 'social participation'/de OR fitness/de OR ((qualit* NEAR/3 life*) OR hrql OR hrqol OR ((health OR functional* OR physic*) NEAR/3 (stat* OR limitation* OR outcome* OR recover* OR impair* OR result* OR fitness*)) OR disab* OR invalid* OR ((walk* OR work* OR mobilit*) NEAR/3 (difficult* OR limit*)) OR (work NEAR/3 (resum* OR return OR back)) OR ((sexual* OR disease* OR assess* OR hand OR adaptat*) NEAR/3 function*) OR (dail* NEAR/3 (life OR living) NEAR/3 activit*) OR adl OR adls OR iadl OR iadls OR badl OR badls OR ((chronic* OR long*-term* OR longterm*) NEAR/3 (pain*)) OR (loss NEAR/3 function*) OR (burn NEAR/3 outcome* NEAR/6 questionnaire*) OR boq OR ((burn OR burns) NEAR/6 health NEAR/3 scale*) OR bshs OR euroqol OR sf36 OR sf-36 OR short-form-36 OR eq-5d OR eq-6d OR eq5d OR eq6d OR itqol OR (physical* NEAR/3 (activ* OR perform*)) OR (independent* NEAR/3 living) OR (social* NEAR/3 participat*)):ab,ti) AND ('cohort analysis'/exp OR 'longitudinal study'/exp OR 'retrospective study'/exp OR 'prospective study'/exp OR 'validation study'/exp OR 'validity'/exp OR 'sensitivity and specificity'/exp OR 'clinical article'/de OR 'observational study'/de OR 'major clinical study'/de OR 'follow up'/de OR 'epidemiological data'/de OR 'case control study'/exp OR 'cross-sectional study'/exp OR (cohort* OR longitudinal* OR retrospectiv* OR prospectiv* OR validat* OR validit* OR (observation* NEAR/3 stud*) OR sensitiv* OR specific* OR 'follow* up*' OR followup* OR epidemiolog* OR 'case control*' OR 'cross-section*'):ab,ti) NOT ([animals]/lim NOT [humans]/lim) NOT ([Conference Abstract]/lim OR [Letter]/lim OR [Note]/lim OR [Editorial]/lim) AND [english]/lim

Medline Ovid

(exp "burns"/ OR "burn units"/ OR (((burn OR burned ) NOT burn*-out*) OR burns OR ((thermal OR heat) ADJ3 injur*)).ab,ti.) AND ("Quality of Life"/ OR "health status"/ OR "Health Status Indicators"/ OR "Disability Evaluation"/ OR "Return to Work"/ OR exp "Activities of Daily Living"/ OR "chronic pain"/ OR "Disabled Persons"/ OR "Motor Activity"/ OR "Physical Fitness"/ OR ((qualit* ADJ3 life*) OR hrql OR hrqol OR ((health OR functional* OR physic*) ADJ3 (stat* OR limitation* OR outcome* OR recover* OR impair* OR result* OR fitness*)) OR disab* OR invalid* OR ((walk* OR work* OR mobilit*) ADJ3 (difficult* OR limit*)) OR (work ADJ3 (resum* OR return OR back)) OR ((sexual* OR disease* OR assess* OR hand OR adaptat*) ADJ3 function*) OR (dail* ADJ3 (life OR living) ADJ3 activit*) OR adl OR adls OR iadl OR iadls OR badl OR badls OR ((chronic* OR long*-term* OR longterm*) ADJ3 (pain*)) OR (loss ADJ3 function*) OR (burn ADJ3 outcome* ADJ6 questionnaire*) OR boq OR ((burn OR burns) ADJ6 health ADJ3 scale*) OR bshs OR euroqol OR sf36 OR sf-36 OR short-form-36 OR eq-5d OR eq-6d OR eq5d OR eq6d OR itqol OR (physical* ADJ3 (activ* OR perform*)) OR (independent* ADJ3 living) OR (social* ADJ3 participat*)).ab,ti.) AND ("validation studies"/ OR "Sensitivity and Specificity"/ OR "observational study"/ OR exp "Epidemiologic Studies"/ OR (cohort* OR longitudinal* OR retrospectiv* OR prospectiv* OR validat* OR validit* OR (observation* ADJ3 stud*) OR sensitiv* OR specific* OR "follow* up*" OR followup* OR epidemiolog* OR "case control*" OR "cross-section*").ab,ti.) NOT (exp animals/ NOT humans/) NOT (letter OR news OR comment OR editorial OR congresses OR abstracts).pt. AND english.la.

CINAHL EBSCOhost

(MH "burns+" OR MH "burn units" OR TI (((burn OR burned ) NOT burn*-out*) OR burns OR ((thermal OR heat) N2 injur*)) OR AB (((burn OR burned ) NOT burn*-out*) OR burns OR ((thermal OR heat) N2 injur*))) AND ("Quality of Life+" OR MH "health status" OR MH "Health Status Indicators" OR MH "Disability Evaluation" OR MH "Job Re-Entry" OR MH "Activities of Daily Living+" OR MH "chronic pain" OR MH "Disabled+" OR MH "Motor Activity" OR MH "Physical Fitness" OR TI ((qualit* N2 life*) OR hrql OR hrqol OR ((health OR functional* OR physic*) N2 (stat* OR limitation* OR outcome* OR recover* OR impair* OR result* OR fitness*)) OR disab* OR invalid* OR ((walk* OR work* OR mobilit*) N2 (difficult* OR limit*)) OR (work N2 (resum* OR return OR back)) OR ((sexual* OR disease* OR assess* OR hand OR adaptat*) N2 function*) OR (dail* N2 (life OR living) N2 activit*) OR adl OR adls OR iadl OR iadls OR badl OR badls OR ((chronic* OR long*-term* OR longterm*) N2 (pain*)) OR (loss N2 function*) OR (burn N2 outcome* N5 questionnaire*) OR boq OR ((burn OR burns) N5 health N2 scale*) OR bshs OR euroqol OR sf36 OR sf-36 OR short-form-36 OR eq-5d OR eq-6d OR eq5d OR eq6d OR itqol OR (physical* N2 (activ* OR perform*)) OR (independent* N2 living) OR (social* N2 participat*)) OR AB ((qualit* N2 life*) OR hrql OR hrqol OR ((health OR functional* OR physic*) N2 (stat* OR limitation* OR outcome* OR recover* OR impair* OR result* OR fitness*)) OR disab* OR invalid* OR ((walk* OR work* OR mobilit*) N2 (difficult* OR limit*)) OR (work N2 (resum* OR return OR back)) OR ((sexual* OR disease* OR assess* OR hand OR adaptat*) N2 function*) OR (dail* N2 (life OR living) N2 activit*) OR adl OR adls OR iadl OR iadls OR badl OR badls OR ((chronic* OR long*-term* OR longterm*) N2 (pain*)) OR (loss N2 function*) OR (burn N2 outcome* N5 questionnaire*) OR boq OR ((burn OR burns) N5 health N2 scale*) OR bshs OR euroqol OR sf36 OR sf-36 OR short-form-36 OR eq-5d OR eq-6d OR eq5d OR eq6d OR itqol OR (physical* N2 (activ* OR perform*)) OR (independent* N2 living) OR (social* N2 participat*))) AND (MH "validation studies" OR MH "Sensitivity and Specificity" OR MH "Nonexperimental Studies" OR MH "Epidemiological Research+" OR TI (cohort* OR longitudinal* OR retrospectiv* OR prospectiv* OR validat* OR validit* OR (observation* N2 stud*) OR sensitiv* OR specific* OR "follow* up*" OR followup* OR epidemiolog* OR "case control*" OR "cross-section*") OR AB (cohort* OR longitudinal* OR retrospectiv* OR prospectiv* OR validat* OR validit* OR (observation* N2 stud*) OR sensitiv* OR specific* OR "follow* up*" OR followup* OR epidemiolog* OR "case control*" OR "cross-section*")) NOT (MH animals+ NOT MH humans+) NOT PT (letter OR news OR comment OR editorial OR congresses OR abstracts) AND LA english

Cochrane

((((burn OR burned ) NOT burn*-out*) OR burns OR ((thermal OR heat) NEAR/3 injur*)):ab,ti) AND (((qualit* NEAR/3 life*) OR hrql OR hrqol OR ((health OR functional* OR physic*) NEAR/3 (stat* OR limitation* OR outcome* OR recover* OR impair* OR result* OR fitness*)) OR disab* OR invalid* OR ((walk* OR work* OR mobilit*) NEAR/3 (difficult* OR limit*)) OR (work NEAR/3 (resum* OR return OR back)) OR ((sexual* OR disease* OR assess* OR hand OR adaptat*) NEAR/3 function*) OR (dail* NEAR/3 (life OR living) NEAR/3 activit*) OR adl OR adls OR iadl OR iadls OR badl OR badls OR ((chronic* OR long*-term* OR longterm*) NEAR/3 (pain*)) OR (loss NEAR/3 function*) OR (burn NEAR/3 outcome* NEAR/6 questionnaire*) OR boq OR ((burn OR burns) NEAR/6 health NEAR/3 scale*) OR bshs OR euroqol OR sf36 OR sf-36 OR short-form-36 OR eq-5d OR eq-6d OR eq5d OR eq6d OR itqol OR (physical* NEAR/3 (activ* OR perform*)) OR (independent* NEAR/3 living) OR (social* NEAR/3 participat*)):ab,ti) AND ((cohort* OR longitudinal* OR retrospectiv* OR prospectiv* OR validat* OR validit* OR (observation* NEAR/3 stud*) OR sensitiv* OR specific* OR 'follow* up*' OR followup* OR epidemiolog* OR 'case control*' OR 'cross-section*'):ab,ti)

Web of science

TS=(((((burn OR burned ) NOT burn*-out*) OR burns OR ((thermal OR heat) NEAR/2 injur*))) AND (((qualit* NEAR/2 life*) OR hrql OR hrqol OR ((health OR functional* OR physic*) NEAR/2 (stat* OR limitation* OR outcome* OR recover* OR impair* OR result* OR fitness*)) OR disab* OR invalid* OR ((walk* OR work* OR mobilit*) NEAR/2 (difficult* OR limit*)) OR (work NEAR/2 (resum* OR return OR back)) OR ((sexual* OR disease* OR assess* OR hand OR adaptat*) NEAR/2 function*) OR (dail* NEAR/2 (life OR living) NEAR/2 activit*) OR adl OR adls OR iadl OR iadls OR badl OR badls OR ((chronic* OR long*-term* OR longterm*) NEAR/2 (pain*)) OR (loss NEAR/2 function*) OR (burn NEAR/2 outcome* NEAR/5 questionnaire*) OR boq OR ((burn OR burns) NEAR/5 health NEAR/2 scale*) OR bshs OR euroqol OR sf36 OR sf-36 OR short-form-36 OR eq-5d OR eq-6d OR eq5d OR eq6d OR itqol OR (physical* NEAR/2 (activ* OR perform*)) OR (independent* NEAR/2 living) OR (social* NEAR/2 participat*))) AND ((cohort* OR longitudinal* OR retrospectiv* OR prospectiv* OR validat* OR validit* OR (observation* NEAR/2 stud*) OR sensitiv* OR specific* OR "follow* up*" OR followup* OR epidemiolog* OR "case control*" OR "cross-section*")) ) AND LA=(english) AND DT=(article)

Google scholar

burn|burns hrql|hrqol|"health|functional|physical status|limitation|outcome|recovery|impairment|fitness"|disability|adl|"chronic pain"|"loss * function" cohort|longitudinal|retrospective|prospective|validation|observational|"follow up"|epidemiological
