# Supplementary material for: Health related quality of life in adults after burn injuries: A systematic review
Source: PLoS One. 2018 May 24;13(5):e0197507. doi: 10.1371/journal.pone.0197507 (PMC5967732; doi:10.1371/journal.pone.0197507)
Supplement: S1 Table — 1Study population: n = sample size; M = males; NA = not applicable. 215D = 15-dimensional health-related quality of life instrument, ALLTAGSLEBEN = multidimensional German questionnaire "every-day-life", BSHS = Burn-specific Health Scale, BSHS-A = Burn-specific Health Scale—Abbreviated, BSHS-B = Burn-specific Health Scale—Brief, BSHS-R = Burn-specific Health Scale—Revised, BSHS-RBA = Burn-specific Health Scale Revised, Brief and Adapted, DLQI = Dermatology Life Quality Index, EQ-5D = EuroQol five dimensions questionnaire, RAND-36 = RAND 36-item health survey, SIP = Sickness Impact Profile, SF-8 = Medical Outcome Study Short Form—8 items, SF-10 = Medical Outcome Study Short Form—10 items, SF-12 = Medical Outcome Study Short Form—12 items, SF-36 = Medical Outcome Study Short Form—36 items, QLQ = Quality of Life Questionnaire, QOLS = quality of life scale, WHODAS = World Health Organization Disability Assessment Schedule, WHOQOL-BREF = World Health Organization Quality of Life—BREF, YABOQ = Young Adult Burn Outcome Questionnaire. (DOCX) [file pone.0197507.s003.docx]

**S1 Table. Characteristics of studies**

| **First author, year (reference)** | **Country** | **Study population^1^** | **%TBSA burned, mean (SD)** | **HRQL instrument(s)^2^** | **Assessment time point(s)** |
| --- | --- | --- | --- | --- | --- |
| Ahuja 2016 [54] | India | n= 60, (M: 40 %).  Median age: 28yr | Median: 30% | BSHS-RBA | Median: 10 months |
| Altier 2002 [23] | Canada | n= 49, (M: 82%).  Mean age: 43yr | 34,59 (13,4) | SF-36 | Mean: 64 months (SD: 28 months) |
| Anzarut 2005 [55] | Canada | n= 47, (M: 96%).  Mean age: 28yr | 64% (2) | BSHS-A, SF-36 | ≥2 years after discharge |
| Baker 2007 [57] | USA | n= 83, (M: 63%).  Mean age: 21yr | 52% (20) | SF-36, QLQ | ≥2 years after discharge |
| Baker 2008 [56] | USA | n= 80, (M: 53%).  Mean age: 21yr | 52% (19) | SF-36, QLQ | ≥2 years after discharge |
| Blades 1979 [17] | USA | n= 32, (M: NA).  Mean age: 43yr | 30% | BSHS | Mean: 9 months, range: 5 – 26 months |
| Blades 1982 [16] | USA | n= 39, (M: NA).  Mean age: 32yr | 26% | Selfmade scale | Mean: 8 months, range: 1 – 25 months |
| Blalock 1994 [101] | USA | n= 254, (M: 74%).  Mean age: 39yr | 19% (15) | BSHS | Mean: 8-9 months |
| Cakir 2015 [18] | Turkey | n= 21, (M: NA).  Mean age: 40yr | 29% | SF-36 | NA |
| Cochran 2004 [21] | USA | n= 32, (M: 94%).  Mean age: 37yr | 5% | SF-36 | ≥1 years after discharge |
| Corry 2010 [106] | USA | n= 171, (M: 70%).  Mean age: 42yr | 15% (13) | SF-36 | Discharge, 1, 6, 12, and 24 months |
| Cromes 2002 [24] | USA | n= 110, (M: 84%).  Mean age: 38yr | 24% | BSHS | 2, 6 and 12 months |
| Daigeler 2009 [58] | Germany | n= 11, (M: 73%).  Mean age: 32yr | 22% | SF-36 | Mean: 6 yr, range: 14 - 118 months |
| Dowda 2014 [25] | Australia | n= 24, (M: 96%).  Mean age: 43yr | 21% | BSHS-B | Discharge, 3, 6, 12 and 24 months |
| Druery 2005 [59] | Australia | n= 38, (M: 88%).  Mean age: 34yr | 61% | BSHS-A | Mean: 6.4 yr |
| Dyster-Aas 2004 [60] | Sweden | n= 86, (M: NA).  Mean age: 44yr | 24% (18) | BSHS-B | Mean 9.0yr (SD: 4.6yr) |
| Dyster-Aas 2007 [19] | Sweden | n= 48, (M: 77%).  Mean age: 41yr | 18% (15) | BSHS-B, SF-36 | Mean 3.6yr (SD: 1.1yr) |
| Edgar 2010 [26] | Australia | n= 280, (M: 81%).  Mean age: 37yr | 9% (12) | BSHS-B, SF-36 | 1, 3, 6, 12 and 24 months |
| Edgar 2013 [27] | Australia | n=1051, (M: 80%).  Mean age: 37yr | 8% (11) | BSHS-B, SF-36 | 1, 3, 6, 12 and 24 months |
| Ekeblad 2015 [61] | Sweden | n=107, (M: 75%).  Mean age: 43yr | 23% | BSHS-B, EQ-5D, SF-36 | 12 months |
| Elsherbiny 2011 [62] | Egypt | n= 100, (M: 38%).  Age: 21-60yr | ≥25% | BSHS-B | NA |
| Fauerbach 1999 [28] | USA | n= 86, (M: 78%).  Mean age: 42yr | 17% (18) | SF-36 | Admission, 2 months |
| Fauerbach 2005 [29] | USA | n= 162, (M: 71%).  Mean age: 41yr | 18% (16) | SF-36 | Preburn, admission, 6 and 12 months |
| Ferreira 2008 [63] | Brazil | n= 115, (M: 66%).  Mean age: 32yr | 19% (16) | BSHS-R | NA |
| Finlay 2009 [30] | Australia | n= 82, (M: 81%).  Mean age: 32yr | 3% (2) | BSHS-B | Admission, 1, 3 and 6 months |
| Gandolfi 2016 [64] | France | n= 53, (M: 66%).  Mean age: 46yr | 27% (16) | BSHS-B, SF-36 | 2 -4 yr post burn |
| Goncalves 2016 [102] | Brazil | n= 108, (M: 68%).  Mean age: 37yr | 13% | BSHS-R | 6 and 12 months |
| Grisbook 2012 [14] | Australia | n= 9, (M: 89%).  Mean age: 39yr | 42% (18) | BSHS-B, SF-36 | Mean: 6.6 yr (SD: 3.7yr) |
| Hashemi 2014 [103] | Iran | n= 110, (M: 63%).  Mean age: 27yr | 26% | BSHS-B | Before intervention, 1 and 2 months after intervention |
| Hwang 2016 [65] | Taiwan | n= 108, (M: 64%).  Mean age: 42yr | 23% (25) | BSHS-B, SF-36 | Mean: 19 months |
| Jarrett 2008 [31] | Australia | n= 86, (M: 86%).  Mean age: 38yr | 11% (16) | SF-36 | Admission, discharge, 1, 3, 6, and 12 months |
| Jonsson 1996 [66] | Sweden | n= 39, (M: 64%).  Median age: 47yr | Median: 8% | SF-36 | 12 months |
| Kildal 2002 [67] | Sweden | n= 248, (M: 80%).  Mean age: 37yr | 23% (16) | BSHS-B | Mean: 9.3yr (SD: 4.8yr) |
| Kildal 2004 [68] | Sweden | n=166, (M: 80%).  Mean age: 50yr | 25% (16) | BSHS-B | Mean: 11.4yr (SD: 4.5yr) |
| Kildal 2005 [69] | Sweden | n=161, (M: 79%).  Mean age: 48yr | 24% (16) | BSHS-B | Mean: 9.2yr (SD: 4.8yr) |
| Kimmo 1998 [70] | Finland | n= 124, (M: 75%).  Mean age: 37yr | 13% (15) | BSHS | Range: 9 months – 7 years |
| Klein 2011 [32] | USA | n= 737, (M: 65%).  Mean age: 70yr | 23% (20) | SF-36 | Baseline, discharge, 6, 12 and 24 months |
| Knight 2017 [71] | Australia | n=41, (M: 81%).  Mean age: 45yr | 8% | BSHS-B | Range: 12-24 months |
| Koljonen 2013 [51] | Finland | n= 44, (M: 59%).  Mean age: 38yr |  | 15D | Discharge, 6, 12 and 24 months |
| Koljonen 2013 [53] | Finland | n= 90, (M: 70%).  Mean age: 46yr |  | 15D | Mean: 23 months (SD: 3 months) |
| Leblebici 2006 [72] | Turkey | n= 22, (M: 64%).  Mean age: 25yr | 28% (17) | SF-36 | Mean: 21 months (SD: 15 months) |
| Li 2014 [73] | China | n= 457, (M: 70%).  Mean age: 37yr | 40% (27) | BSHS-A, EQ-5D, SF-36 | Mean: 14 months (SD: 1 months) |
| Ling-Juan 2012 [107] | China | n= 208, (M: 77%).  Mean age: 40yr | 40% (27) | BSHS-B | Range: 1 – 137 months |
| Low 2012 [74] | Sweden | n=85, (M: 75%).  Mean age: 45yr | 24% (20) | BSHS-B | 12 months |
| Mazharinia 2007 [75] | Iran | n= 109, (M: 44%).  Mean age: 29yr | NA | DLQI | NA |
| Meirte 2016 [76] | The Netherlands | n= 184, (M: 71%).  Mean age: 39yr | 12% (10) | BSHS-B, EQ-5D, SF-36 | 9 months |
| Miller 2013 [15] | USA | n= 1547, (M: NA).  Mean age: NA | 20% | SF-36, SF-12, SF-10 | Preburn, admission, 6, 12 and 24 months |
| Moi 2003[78] | Norway | n= 95, (M: 82%).  Mean age: 44yr | 19% (14) | BSHS-A, SF-36 | Mean: 47 months (SD: 24 months) |
| Moi 2006 [80] | Norway | n= 95, (M: 82%).  Mean age: 44yr | 19% (14) | SF-36, QOLS | Mean: 47 months (SD: 24 months) |
| Moi 2007 [79] | Norway | n= 95, (M: 82%).  Mean age: 44yr | 19% (14) | BSHS-A | Mean: 47 months (SD: 24 months) |
| Moi 2012 [77] | Norway | n= 95, (M: 82%).  Mean age: 44yr | 19% (14) | BSHS-A, SF-36, QOLS | Mean: 47 months (SD: 24 months) |
| Mulay 2015 [81] | India | n= 20, (M: 40%).  Mean age: 31yr | 40% | BSHS-B, BSHS-RBA | Range: 6 – 12 months |
| Müller 2015 [82] | Germany | n= 141, (M: 65%).  Mean age: 50yr | 13% (10) | BSHS-B, SF-8 | Mean: 45 months (SD: 26 months) |
| Munster 1996 [20] | USA | n= NA, (M: NA).  Mean age: NA | NA | BSHS | NA |
| Murphy 2015 [83] | USA | n= 50, (M: 56%).  Mean age: 18yr | 50% (22) | BSHS-B, WHODAS | Range: 2.5 – 12.5 yr post burn |
| Niţescu 2012 [33] | Romania | n= 26, (M: 58%).  Age: 21-57yr | >25% | WHOQOL-BREF | Admission, 3, 6, 12 months |
| Noble 2006 [84] | Canada | n= 22, (M: 96%).  Mean age: 44yr | 16% (12) | BSHS-B | Mean: 5.3 yr (4.9yr) |
| Novelli 2009 [34] | Italy | n= 30, (M: 60%).  Mean age: 42yr | 32% (13) | SIP | Discharge, 3 months |
| Orwelius 2013 [35] | Sweden | n= 156, (M: 74%).  Mean age: 46yr | 24% (19) | SF-36 | 12 and 24 months |
| Öster 2009 [36] | Sweden | n= 78, (M: 78%).  Mean age: 44yr | 24% (20) | BSHS-B, EQ-5D, SF-36 | Admission, 3, 6, 12 months |
| Öster 2011 [37] | Sweden | n= 89, (M: 77%).  Mean age: 43yr | 25% (20) | EQ-5D | Admission, 3, 6, 12 months and 2 to 7 yr |
| Öster 2013 [38] | Sweden | n= 67, (M: 78%).  Mean age: 43yr | 25% (20) | BSHS-B | 6, 12 months and 2 to 7 yr |
| Pallua 2003 [85] | Germany | n= 92, (M: 74%).  Mean age: 40yr | 21% (15) | ALLTAGSLEBEN | Mean : 5.4yr (SD: 1.1yr) |
| Palmieri 2012 [86] | USA | n= 232, (M: 62%).  Mean age: 72yr | 8% | SF-12 | Mean: 46 months |
| Palmu 2015 [39] | Finland | n= 92, (M: 70%).  Mean age: 46yr | 10% | 15D, EQ-5D, RAND-36 | 6 months |
| Pavoni 2010 [87] | Italy | n= 19, (M: 58%).  Mean age: 46yr | 50% (16) | EQ-5D | 12 months |
| Pfitzer 2016 [40] | Australia | n= 13, (M: 62%).  Mean age: 54yr | 16% (11) | BSHS-B, SF-36 | preburn, 3, 6, 12, 36 months |
| Piccolo 2015 [88] | Brazil | n= 92, (M: 52%).  Mean age: 37yr | 19% (19) | BSHS-B, BSHS-R | Mean: 4.4yr (SD: 4.7yr) |
| Pishnamazi 2013 [89] | Iran | n= 200, (M: 38%).  Mean age: 25yr | 35% (2) | BSHS-B | NA |
| Renneberg 2014 [41] | Germany | n= 265, (M: 72%).  Mean age: 39yr | 14% (14) | BSHS-B, SF-12 | Admission, 6, 12, 24, and 36 months |
| Ricci 2014 [90] | Brazil | n= 73, (M: 69%).  Mean age: 38yr | 14% (12) | BSHS-R | 5 to 7 months |
| Roh 2012[91] | South Korea | n= 113, (M: 71%).  Mean age: 38yr | 14% (12) | BSHS-B | Mean: 1 month |
| Rosenberg 2006 [92] | USA | n= 95, (M: 55%).  Mean age: 21yr | 54% (20) | QLQ | Mean: 14.2 yr (5.5yr) |
| Rosenberg 2015 [93] | USA | n= 123, (M: 64%).  Mean age: 20yr | 49% | BSHS-B | Inhalation group: mean 8,0yr (SD: 3,1yr), non-inhalation: mean: 9,1yr (SD: 3,0yr) |
| Ryan 2013 [43] | USA | n= 153, (M: 73%).  Mean age: 25yr | 11% (14) | YABOQ | Admission, 2 weeks, 6 and 12 months |
| Ryan 2015 [42] | USA | n= 147, (M: 72%).  Mean age: 25yr | 11% | YABOQ | Admission, 2 weeks, 6 and 12 months |
| Salvador Sanz 1998 [94] | Spain | n= 115, (M: 54%).  Mean age: 41yr | 14% (12) | BSHS | Mean: 26 months |
| Salvador Sanz 1999 [95] | Spain | n= 115, (M: 54%).  Mean age: 41yr | 14% (12) | BSHS | Mean: 26 months |
| Stavrou 2015 [105] | Israel | n= 86, (M: 79%).  Mean age: 38yr | 11% (12) | BSHS-B, SF-36 | Mean: 12 months (SD: 13 months) |
| Szczechowicz 2014 [96] | Poland | n= 50, (M: 66%).  Mean age: 47yr | 24% (16) | BSHS-B, SF-36 | NA |
| Tahir 2011 [44] | Pakistan | n=99, (M: 68%).  Median age: 30yr | 19% | SF-36 | Admission, 5 and 6 months |
| Tang 2015 [104] | China | n= 55, (M: 80%).  Mean age: 39yr | NA | WHOQOL-BREF | Rehabilitation group: mean: 3,97 months (SD: 2,30), conventional care group: mean: 3,12 months (SD: 2,32) |
| Van Loey 2012 [45] | The Netherlands and Belgium | n= 244, (M: 73%).  Mean age: 39yr | 12% (11), range 1-65% | EQ-5D | 3 weeks, 3, 9 and 18 months |
| Wasiak 2013 [48] | Australia | n= 15, (M: 67%).  Mean age: 51yr | 18% (13) | BSHS-B, SF-36 | Preburn, 3, 6 and 12 months |
| Wasiak 2014 [46] | Australia | n= 99, (M: 75%).  Mean age: 42yr | 19% | BSHS-B, SF-36 | Preburn and 12 months |
| Wasiak 2014 [49] | Australia | n= 114, (M: 75%).  Mean age:41yr | 17% | BSHS-B, SF-36 | Preburn, 3, 6 and 12 months |
| Wasiak 2016 [47] | Australia | n= 114, (M: 75%).  Mean age: 41yr | 17% | BSHS-B, SF-36 | Preburn, 3, 6 and 12 months |
| Willebrand 2006 [97] | Sweden | n= 86, (M: 73%).  Mean age: 43yr | 17% (14) | BSHS-B | Mean: 3.6yr (SD: 1.2yr) |
| Willebrand 2011 [52] | Sweden | n= 94, (M: 76%).  Mean age: 44yr | 23% (20) | BSHS-B, SF-36 | 6, 12 and 24 months |
| Williams 2012 [50] | Australia | n= 52, (M: 70%).  Mean age: 39yr | NA | BSHS-B | Admission, 1, 3,6 and 12 months |
| Xie 2012 [100] | China | n= 103, (M: 83%).  Mean age: 40yr | 88% | BSHS-B | Mean: 3.0 yr |
| Xie 2012 [22] | China | n= 20, (M: 70%).  Mean age: 43yr | 84% (10) | BSHS-B, SF-36 | ≥2 years after discharge |
| Zhang 2014 [98] | China | n= 208, (M: 77%).  Mean age: 42yr | 42% (27) | BSHS-B | ≥2 years after discharge |
| Zorita 2016 [99] | Brazil | n= 30, (M: 57%).  Mean age: 36yr | 21% (17) | SF-36 | Mean: 15 months, range: 6-24 months |

^1^Study population: n=sample size; M= males; NA= not applicable
^2^15D = 15-dimensional health-related quality of life instrument, ALLTAGSLEBEN = multidimensional German questionnaire "every-day-life", BSHS = Burn-specific Health Scale, BSHS-A = Burn-specific Health Scale - Abbreviated, BSHS-B = Burn-specific Health Scale - Brief, BSHS-RBA = Burn-specific Health Scale Revised, Brief and Adapted, DLQI = Dermatology Life Quality Index, EQ-5D = EuroQol five dimensions questionnaire, RAND-36 = RAND 36-item health survey, SIP = Sickness Impact Profile, SF-8 = Medical Outcome Study Short Form - 8 items, SF-10 = Medical Outcome Study Short Form - 10 items, SF-12 = Medical Outcome Study Short Form - 12 items, SF-36 = Medical Outcome Study Short Form - 36 items, QLQ = Quality of Life Questionnaire, QOLS = quality of life scale, WHODAS = World Health Organization Disability Assessment Schedule, WHOQOL-BREF = World Health Organization Quality of Life - BREF, YABOQ = Young Adult Burn Outcome Questionnaire
