# Supplementary material for: Health related quality of life in adults after burn injuries: A systematic review
Source: PLoS One. 2018 May 24;13(5):e0197507. doi: 10.1371/journal.pone.0197507 (PMC5967732; doi:10.1371/journal.pone.0197507)
Supplement: S2 Table — (DOCX) [file pone.0197507.s004.docx]

**S2 Table. Risk of bias assessed with four domains of the Quality in Prognostic Studies (QUIPS) risk of bias tool**

| **First author, year (reference)** | **1. Study Participation** | **2. Study Attrition** | **4. Outcome Measurement** | **6. Statistical Analysis and Reporting** |
| --- | --- | --- | --- | --- |
| Ahuja 2016 [54] | Low risk | Low risk | Low risk | Low risk |
| Altier 2002 [23] | Moderate risk | Low risk | Low risk | Low risk |
| Anzarut 2005 [55] | Moderate risk | Moderate risk | Moderate risk | Moderate risk |
| Baker 2007 [57] | Moderate risk | High risk | Low risk | High risk |
| Baker 2008 [56] | Moderate risk | High risk | Low risk | Low risk |
| Blades 1979 [17] | High risk | High risk | High risk | High risk |
| Blades 1982 [16] | Moderate risk | High risk | Moderate risk | High risk |
| Blalock 1994 [101] | Moderate risk | High risk | Low risk | Low risk |
| Cakir 2015 [18] | Moderate risk | High risk | Low risk | Low risk |
| Cochran 2004 [21] | Moderate risk | High risk | Low risk | Low risk |
| Corry 2010 [106] | Moderate risk | High risk | Low risk | Low risk |
| Cromes 2002 [24] | Moderate risk | High risk | Low risk | Moderate risk |
| Daigeler 2009 [58] | Moderate risk | Low risk | Low risk | Low risk |
| Dowda 2014 [25] | Moderate risk | High risk | Low risk | Moderate risk |
| Druery 2005 [59] | Low risk | High risk | Low risk | Moderate risk |
| Dyster-Aas 2004 [60] | Moderate risk | High risk | Low risk | Low risk |
| Dyster-Aas 2007 [19] | Low risk | Moderate risk | Low risk | Low risk |
| Edgar 2010 [26] | Moderate risk | High risk | Low risk | Low risk |
| Edgar 2013 [27] | Low risk | Low risk | Low risk | Low risk |
| Ekeblad 2015 [61] | Low risk | Moderate risk | Low risk | Low risk |
| Elsherbiny 2011 [62] | High risk | High risk | Low risk | Moderate risk |
| Fauerbach 1999 [28] | Moderate risk | High risk | Low risk | Low risk |
| Fauerbach 2005 [29] | Moderate risk | High risk | Low risk | Moderate risk |
| Ferreira 2008 [63] | Moderate risk | High risk | Low risk | Low risk |
| Finlay 2009 [30] | Low risk | Moderate risk | Low risk | Low risk |
| Gandolfi 2016 [64] | Low risk | High risk | Low risk | Low risk |
| Goncalves 2016 [102] | Low risk | High risk | Low risk | Low risk |
| Grisbook 2012 [14] | Moderate risk | High risk | Low risk | Low risk |
| Hashemi 2014 [103] | Moderate risk | High risk | Low risk | Low risk |
| Hwang 2016 [65] | Moderate risk | High risk | Low risk | Low risk |
| Jarrett 2008 [31] | Moderate risk | High risk | Low risk | Low risk |
| Jonsson 1996 [66] | Moderate risk | High risk | Low risk | Moderate risk |
| Kildal 2002 [67] | Low risk | Moderate risk | Low risk | Low risk |
| Kildal 2004 [68] | Low risk | Moderate risk | Low risk | Low risk |
| Kildal 2005 [69] | Low risk | Moderate risk | Low risk | Low risk |
| Kimmo 1998 [70] | Low risk | High risk | Low risk | Low risk |
| Klein 2011 [32] | Low risk | Low risk | Low risk | Low risk |
| Knight 2016 [71] | Moderate risk | High risk | Low risk | Low risk |
| Koljonen 2013 [51] | Low risk | High risk | Low risk | Low risk |
| Koljonen 2013 [53] | Moderate risk | Moderate risk | Low risk | Low risk |
| Leblebici 2006 [72] | Moderate risk | High risk | Low risk | Low risk |
| Li 2014 [73] | Moderate risk | Moderate risk | Low risk | Low risk |
| Ling-Juan 2012 [107] | Low risk | High risk | Low risk | Low risk |
| Low 2012 [74] | Low risk | Moderate risk | Low risk | Low risk |
| Mazharinia 2007 [75] | Moderate risk | High risk | Low risk | Low risk |
| Meirte 2016 [76] | Low risk | High risk | Low risk | Low risk |
| Miller 2013 [15] | High risk | High risk | Moderate risk | Low risk |
| Moi 2003[78] | Low risk | Moderate risk | Low risk | Low risk |
| Moi 2006 [80] | Low risk | Moderate risk | Low risk | Low risk |
| Moi 2007 [79] | Low risk | Moderate risk | Low risk | Low risk |
| Moi 2012 [77] | Low risk | Moderate risk | Low risk | Low risk |
| Mulay 2015 [81] | Moderate risk | High risk | Low risk | Low risk |
| Müller 2015 [82] | Moderate risk | High risk | Low risk | Low risk |
| Munster 1996 [20] | High risk | High risk | Moderate risk | High risk |
| Murphy 2015 [83] | Moderate risk | High risk | Low risk | Low risk |
| Niţescu 2012 [33] | Low risk | Low risk | Low risk | Low risk |
| Noble 2006 [84] | Moderate risk | High risk | Low risk | Low risk |
| Novelli 2009 [34] | High risk | High risk | Low risk | Moderate risk |
| Orwelius 2013 [35] | Low risk | Moderate risk | Low risk | Low risk |
| Öster 2009 [36] | Low risk | Moderate risk | Low risk | Low risk |
| Öster 2011 [37] | Low risk | Moderate risk | Low risk | Low risk |
| Öster 2013 [38] | Low risk | Moderate risk | Low risk | Low risk |
| Pallua 2003 [85] | Moderate risk | High risk | Moderate risk | Moderate risk |
| Palmieri 2012 [86] | Low risk | High risk | Low risk | Low risk |
| Palmu 2015 [39] | Low risk | Moderate risk | Low risk | Moderate risk |
| Pavoni 2010 [87] | Low risk | Low risk | Low risk | Moderate risk |
| Pfitzer 2016 [40] | Low risk | High risk | Low risk | Low risk |
| Piccolo 2015 [88] | Moderate risk | High risk | Low risk | High risk |
| Pishnamazi 2013 [89] | Moderate risk | High risk | Low risk | Low risk |
| Renneberg 2014 [41] | Moderate risk | High risk | Low risk | Low risk |
| Ricci 2014 [90] | Moderate risk | High risk | Low risk | Low risk |
| Roh 2012[91] | Moderate risk | High risk | Low risk | Low risk |
| Rosenberg 2006 [92] | High risk | High risk | Moderate risk | Low risk |
| Rosenberg 2015 [93] | Moderate risk | High risk | Low risk | Moderate risk |
| Ryan 2013 [43] | Moderate risk | High risk | Low risk | Low risk |
| Ryan 2015 [42] | Moderate risk | High risk | Low risk | Low risk |
| Salvador Sanz 1998 [94] | Moderate risk | Moderate risk | Low risk | Low risk |
| Salvador Sanz 1999 [95] | Moderate risk | Moderate risk | Low risk | Low risk |
| Stavrou 2015 [105] | Moderate risk | High risk | Low risk | Low risk |
| Szczechowicz 2014 [96] | High risk | High risk | Low risk | Low risk |
| Tahir 2011 [44] | Low risk | High risk | Low risk | Moderate risk |
| Tang 2015 [104] | Moderate risk | High risk | Low risk | Low risk |
| Van Loey 2012 [45] | Low risk | High risk | Low risk | Low risk |
| Wasiak 2013 [48] | Low risk | High risk | Low risk | Low risk |
| Wasiak 2014a [46] | Low risk | High risk | Low risk | Low risk |
| Wasiak 2014b [49] | Low risk | High risk | Low risk | Low risk |
| Wasiak 2016 [47] | Low risk | High risk | Low risk | Low risk |
| Willebrand 2006 [97] | Low risk | High risk | Low risk | Low risk |
| Willebrand 2011 [52] | Low risk | Moderate risk | Low risk | Low risk |
| Williams 2012 [50] | Low risk | High risk | Low risk | Moderate risk |
| Xie 2012 [100] | Moderate risk | High risk | High risk | Moderate risk |
| Xie 2012 [22] | Moderate risk | Moderate risk | Low risk | Low risk |
| Zhang 2014 [98] | Low risk | Moderate risk | Low risk | Moderate risk |
| Zorita 2016 [99] | Moderate risk | High risk | Low risk | Low risk |
