# Supplementary figures and images for: Health related quality of life in adults after burn injuries: A systematic review
Source: PLoS One. 2018 May 24;13(5):e0197507. doi: 10.1371/journal.pone.0197507 (PMC5967732; doi:10.1371/journal.pone.0197507)

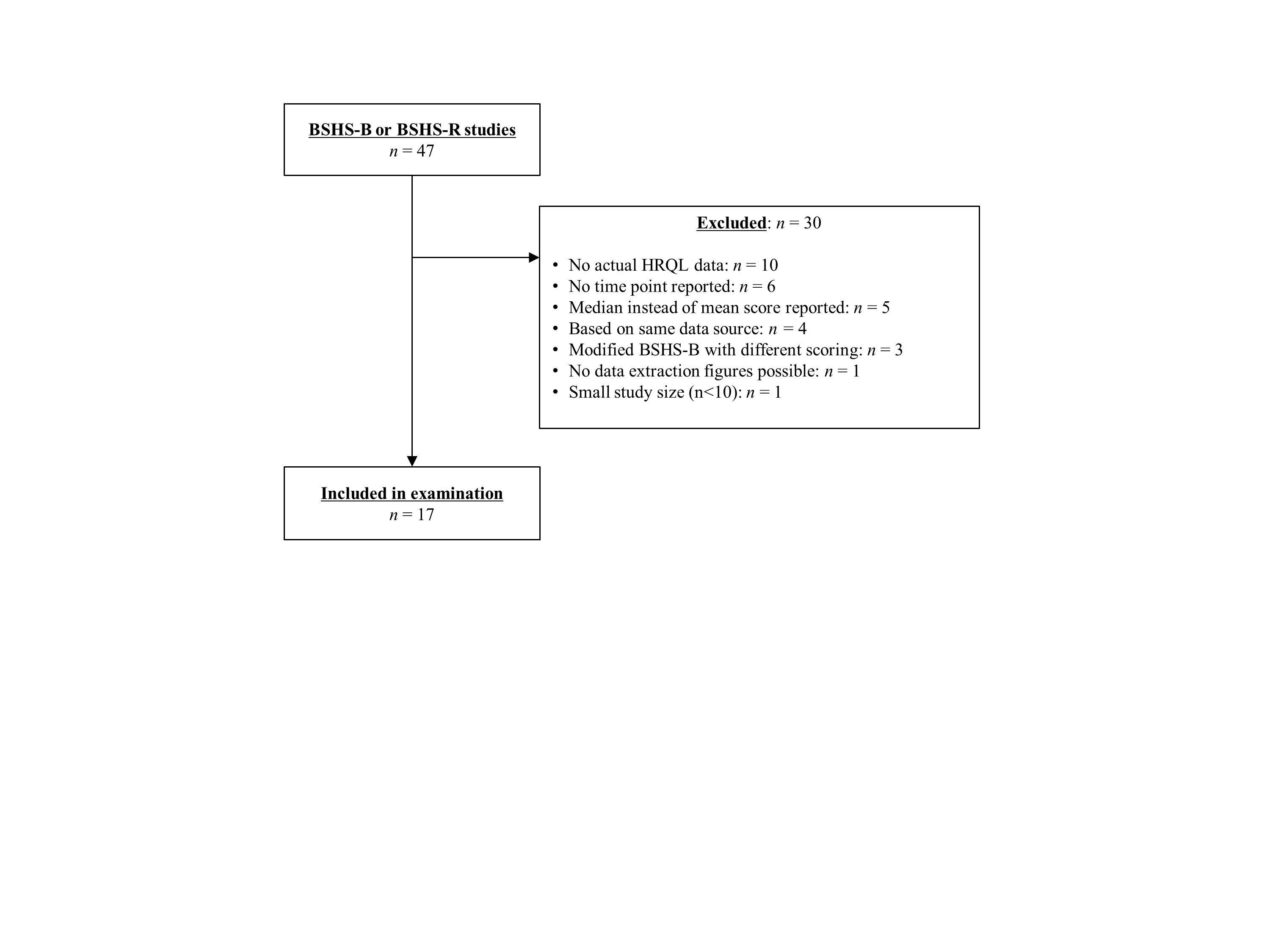

Supplement: S1 Fig — (TIF) [file pone.0197507.s005.tif]

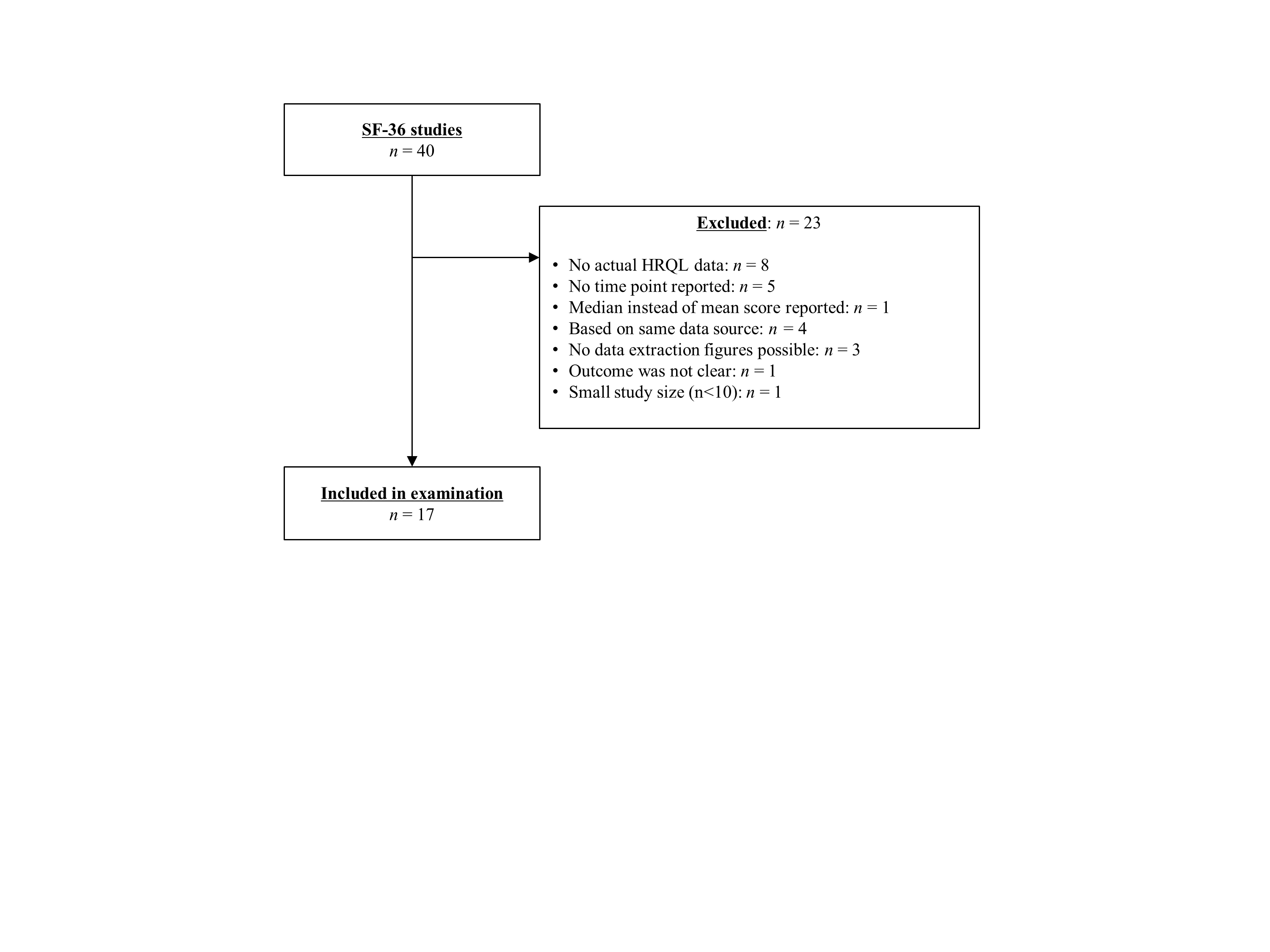

Supplement: S2 Fig — (TIF) [file pone.0197507.s006.tif]
